# Rhino-Sino-Orbital Mucormycosis in Cholangiocarcinoma and Single-Centre Experience with Mucormycosis in Oncohematologic Patients: An Eight-Case Series

**DOI:** 10.3390/jof12100744

**Published:** 2026-10-04

**Authors:** Cătana Alina Camelia, Birlutiu Victoria, Cipăian Călin Remus, Patran Monica, Sandu Mariana, Dobra Dina, Mihai Elena, Oltean Ciprian, Olteanu Ariela, Ercută Vlad, Ștef Laura, Moscu Bianca, Porr Corina, Iancu Gabriela, Mondoc Lidia-Maria

**Affiliations:** 1 County Clinical Emergency Hospital Sibiu, 550245 Sibiu, Romania; alinacamelia.catana@ulbsibiu.ro (C.A.C.);; 2 Faculty of Medicine Sibiu, Lucian Blaga University of Sibiu, Str. Lucian Blaga, Nr. 2A, 550169 Sibiu, Romania

**Keywords:** mucormycosis, cholangiocarcinoma, hematologic malignancies, rhino-sino-orbito-cerebral, pulmonary, urinary mucormycosis, immunocompromised host

## Abstract

**Background:** Mucormycosis is a rapidly progressive and often fatal invasive fungal infection requiring immediate suspicion-driven diagnosis and treatment, particularly in immunocompromised patients. We report the first documented case of rhino-sino-orbital mucormycosis in a patient with recurrent intrahepatic cholangiocarcinoma and summarize our single-centre experience with eight cases of mucormycosis in oncohematologic patients. **Methods:** A 50-year-old woman developed rhino-sino-orbital mucormycosis after multimodal treatment for recurrent cholangiocarcinoma. Because of high clinical suspicion, imaging was performed before microbiological confirmation and antifungal therapy was initiated immediately through multidisciplinary management. **Results:** The patient survived 16 years after the initial cancer diagnosis, seven years after recurrence, and two years following mucormycosis. Our institutional series included eight cases with rhino-sino-orbital, pulmonary, cutaneous, and urinary tract involvement, most diagnosed after 2020, highlighting heterogeneous presentations and breakthrough infection despite prior antifungal exposure. Clinical experience gained from these cases contributed to early recognition and successful management of the present patient. **Conclusions:** To our knowledge, this is the first reported case of mucormycosis in cholangiocarcinoma. These findings emphasize that mucormycosis should be considered a medical emergency in patients with hematologic malignancies or solid tumors, and antifungal therapy should be initiated based solely on clinical suspicion without waiting for microbiological confirmation, even in atypical presentations.

## 1. Introduction

Mucormycosis is a rapidly progressive and often fatal invasive fungal infection that primarily affects immunocompromised patients and requires early recognition and prompt treatment [1,2,3]. Cholangiocarcinoma (CCA) is a rare malignancy of the biliary tract characterized by aggressive behavior, frequent late diagnosis, and poor clinical outcomes [4,5]. Although distant metastatic disease may occur, neuro-ophthalmologic and orbital manifestations are exceptionally uncommon [6,7]. Importantly, invasive fungal disease and biliary malignancy may also present with overlapping clinical and radiological features; hepatic mucormycosis involving the biliary tract has been reported to mimic hilar cholangiocarcinoma [8]. In patients with hematologic malignancies, mucormycosis is a particularly severe opportunistic infection, with pulmonary and rhino-sino-orbital/rhinocerebral involvement among the major clinical presentations and with substantial associated mortality [9,10]. The incidence of mucormycosis has increased in recent years, particularly among patients with hematologic malignancies, solid tumors, recipients of hematopoietic stem cell or solid organ transplantation, and in the context of diabetes mellitus, prolonged neutropenia, immunosuppressive therapies, and SARS-CoV-2 infection [11,12,13,14]. Among its clinical forms, rhino-orbito-cerebral mucormycosis is one of the most severe presentations, often associated with rapid progression and high mortality. In this context, orbital involvement represents a diagnostic challenge, as it may mimic a wide range of conditions, including infectious, inflammatory, and neoplastic processes, particularly in patients with underlying malignancies.

Biliary tract cancers are a heterogeneous group of aggressive malignancies associated with high mortality [15]. These tumors comprise distinct anatomical subtypes, including intrahepatic and extrahepatic cholangiocarcinoma, with differing clinical characteristics and outcomes; ampullary carcinoma has been reported to have a more favorable prognosis compared with extrahepatic cholangiocarcinoma [16,17].

Cholangiocarcinoma most commonly involves regional lymph nodes and adjacent organs, whereas distant metastases are uncommon. Brain metastases represent an exceptionally rare event and have been described predominantly in isolated case reports [18,19]. Orbital and neuro-ophthalmologic manifestations of cholangiocarcinoma are exceptionally rare, with only a limited number of orbital, skull-base, and cranial metastases reported in the literature, predominantly as isolated case reports [6,20,21,22].

Once orbital lesions are identified in patients with an underlying malignancy, careful differential diagnosis is essential, including infectious and inflammatory conditions, benign and malignant tumors, and vascular malformations. Among orbital infections, fungal etiologies are uncommon but increasingly recognized, particularly in immunocompromised patients, including those with hematologic malignancies, as well as recipients of hematopoietic stem cell or solid organ transplantation [1,11,12,13].

Therefore, this study aims to describe a rare case of rhino-sino-orbital mucormycosis in a patient with cholangiocarcinoma, together with seven additional cases of mucormycosis in patients with hematologic malignancies, and to highlight the importance of considering mucormycosis in the differential diagnosis of acute orbital and neurological manifestations in oncological patients.

## 2. Case Presentation

### Detailed Case Description

**Figure 1:**
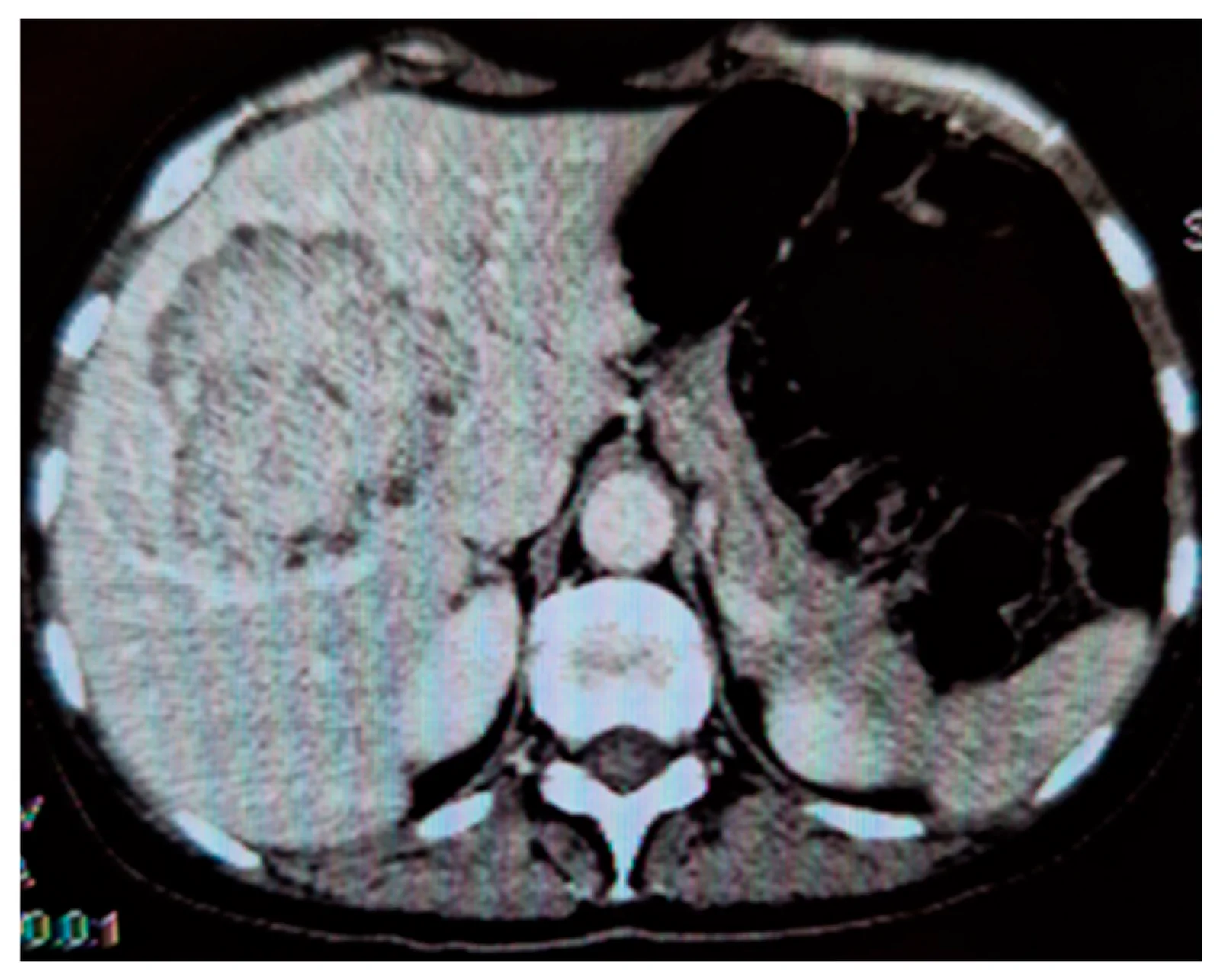
Abdominal CT showing a right hepatic lobe polycystic tumor measuring 8 cm in diameter, with isodense enhancement, peripheral hypodense areas, and central isodense enhancement, exerting a mass effect on the vascular structures.

We report the case of a 50-year-old female patient in whom a liver mass in the right hepatic lobe (RHL) was detected during routine ultrasound examination in July 2009 and subsequently confirmed by computed tomography (CT) and magnetic resonance imaging (MRI). CT revealed a polycystic tumor of the right hepatic lobe, measuring 8 cm in diameter, with isodense areas, peripheral hypodense regions, and central isodense enhancement, exerting a mass effect on the vascular structures, without evidence of locoregional or distant metastases (Figure 1).

**Figure 2:**
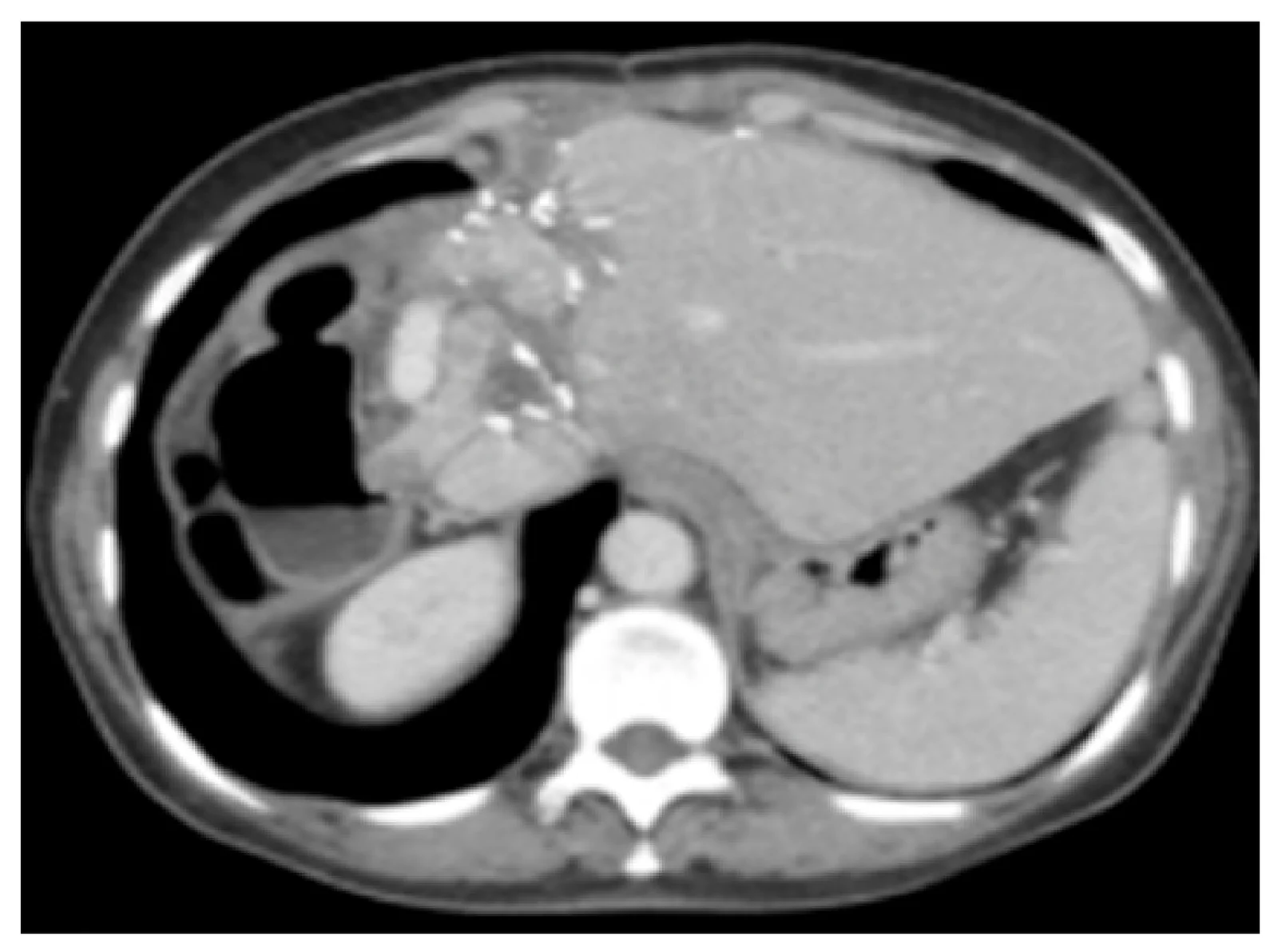
Compensatory hypertrophy of the left hepatic lobe (LHL) and the presence of hepatorrhaphy material following right lobectomy.

An MRI-guided biopsy was performed. Histopathological examination established the diagnosis of intrahepatic cholangiocarcinoma, with TNM classification: G2, pT1, L0, V0. An extended right hemihepatectomy was performed in Regensburg, with a favorable outcome. Postoperatively, compensatory hypertrophy of the left hepatic lobe and the presence of right-sided hepatorrhaphy material were noted following right lobectomy (Figure 2).

**Figure 3:**
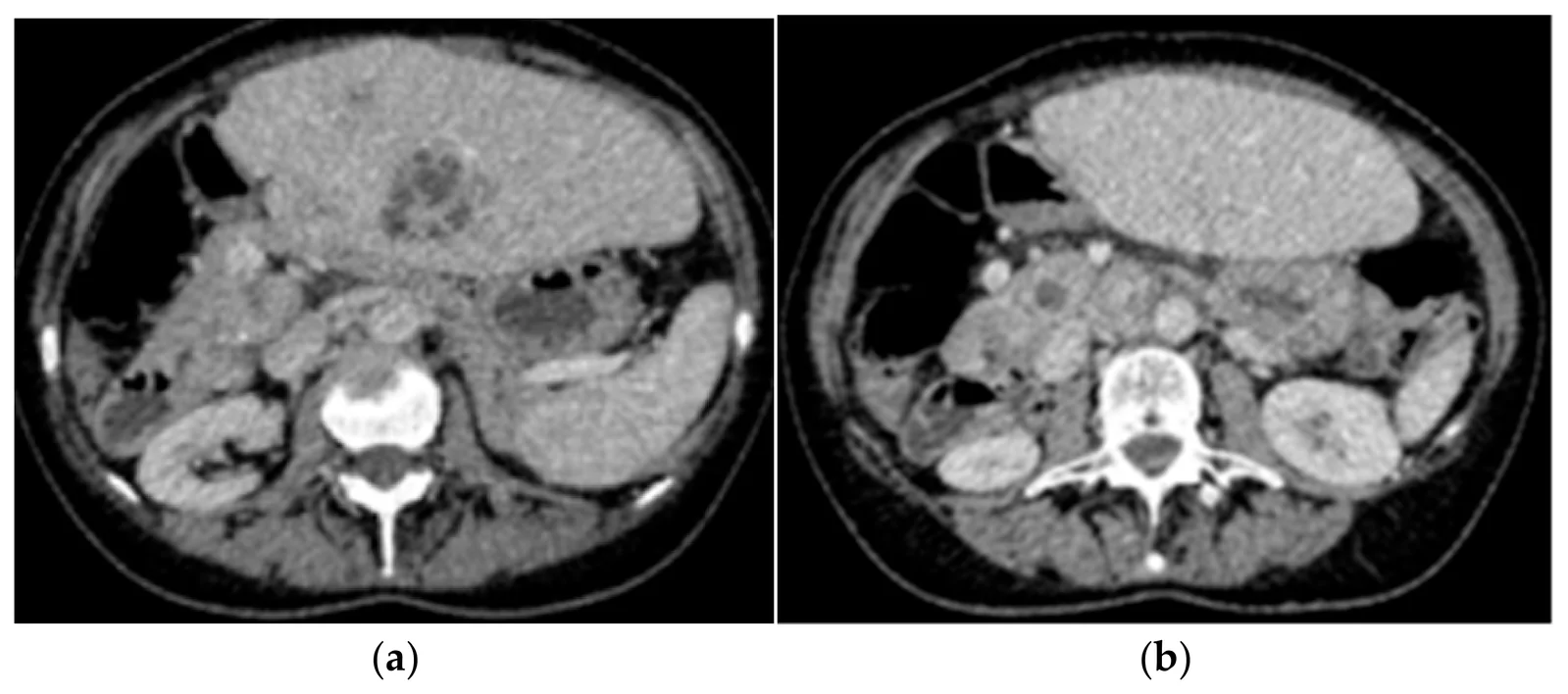
(**a**,**b**) The remaining postoperative left hepatic lobe (LHL) shows a heterogeneous structure with nodular areas suggestive of secondary liver metastases. Paracaval and interaortocaval lymphadenopathy with imaging features similar to those of the hepatic lesions and the lesion at initial diagnosis are also observed.

After a 9-year remission period, in September 2018, recurrence of cholangiocarcinoma in the left hepatic lobe was identified, along with high-grade hepatic and retroperitoneal metastases, documented by MRI and histopathological examination following a percutaneous liver biopsy performed at the Department of Gastroenterology in Cluj (Figure 3).

Six cycles of chemotherapy with gemcitabine and oxaliplatin were administered concurrently with immunogenic cell death (ICD), modulated electrohyperthermia (mEHT), and oncolytic virotherapy with Newcastle disease virus (NDV) in Cologne, Germany. MRI evaluation after 3 and 6 cycles suggested a partial therapeutic response. Molecular investigations and drug sensitivity testing revealed overexpression of the PD-L1 gene, with a certified recommendation for targeted immunotherapy with nivolumab. The patient received nivolumab and gemcitabine concurrently with ICD, mEHT, and oncolytic virotherapy with NDV.

Positron emission tomography–computed tomography (PET–CT) scans performed after 3, 7, and 11 cycles, in June 2019, October 2019, and March 2020, revealed stable hepatic lesions, metabolically inactive residual retroperitoneal lymphadenopathy, and a near-complete metabolic response.

After a 6-month treatment-free interval, in October 2020, CT and PET–CT scans demonstrated progression of hepatic and abdominal lesions. A biopsy of a retroperitoneal lymph node was performed via an ultrasound-guided transabdominal approach, histologically confirming recurrence of cholangiocarcinoma.

Selective intrahepatic radiotherapy (SIRT) was performed in two procedures, two years apart, in November 2018 and 2020, with administration of yttrium-90 microspheres into the left hepatic artery at the Department of Nuclear Medicine, Barmherzige Brüder University Hospital, Regensburg, Germany.

Immunotherapy with the autologous antitumor vaccine IO-VAC was subsequently initiated, with dose 1 administered in August 2022, dose 2 in October 2022, and a booster dose (dose 3) administered six months later, on 10 May 2023, at IOZK, Germany. Treatment with nivolumab at a dose of 140 mg was continued at 3- to 4-week intervals, with the last dose administered on 31 May 2023, two weeks after the IO-VAC booster dose. MEHT, oncolytic virotherapy with NDV, and moderate whole-body hyperthermia (WBH), as well as ozone therapy, Curcumin Direct Spray, and DC vaccine, were administered in combination, with rigorous monitoring of the immune response.

**Figure 4:**
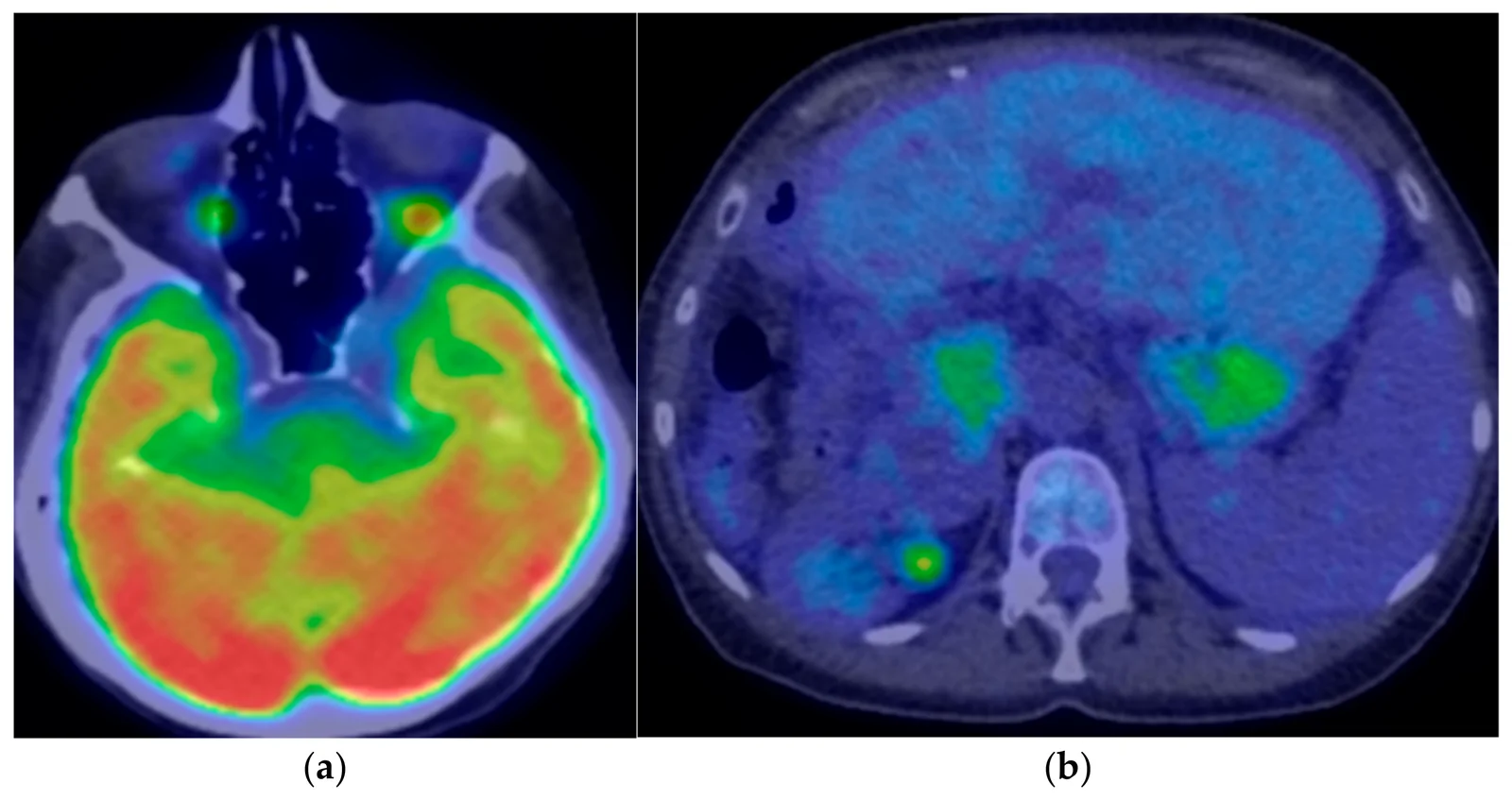
(**a**,**b**) No pathological enhancement is observed in the oculoorbital regions; the liver remains heterogeneous, and several retroperitoneal lymph nodes are present without post-contrast enhancement.

MRI performed in November 2022 showed mild accentuation of internal softening of splenic, hepatic, and lymph node lesions, with no new lesions and no dimensional progression. A PET–CT scan performed in February 2023 revealed retroperitoneal adenopathy with progressive low-avid FDG uptake (Figure 4).

Dental procedures were scheduled for May 2023. Following administration of the autologous antitumor vaccine on 10 May 2023, and nivolumab on 31 May 2023, the patient developed asthenia, abdominal fluid retention, and edema of the lower extremities.

On 24 June 2023, at the age of 64, at 08:00 a.m., the patient presented with marked asthenia, right-sided hemicrania, progressive unilateral right eyelid edema (non-erythematous), conjunctival chemosis, and severe ocular pain on horizontal and vertical eye movements. This was followed by limitation of ocular motility, diplopia, eyelid ptosis with inability to elevate the eyelid, photophobia upon manual elevation of the eyelid, nasolabial swelling, facial asymmetry, and right-sided exophthalmos measuring 19 mm (15 mm on the left).

**Figure 5:**
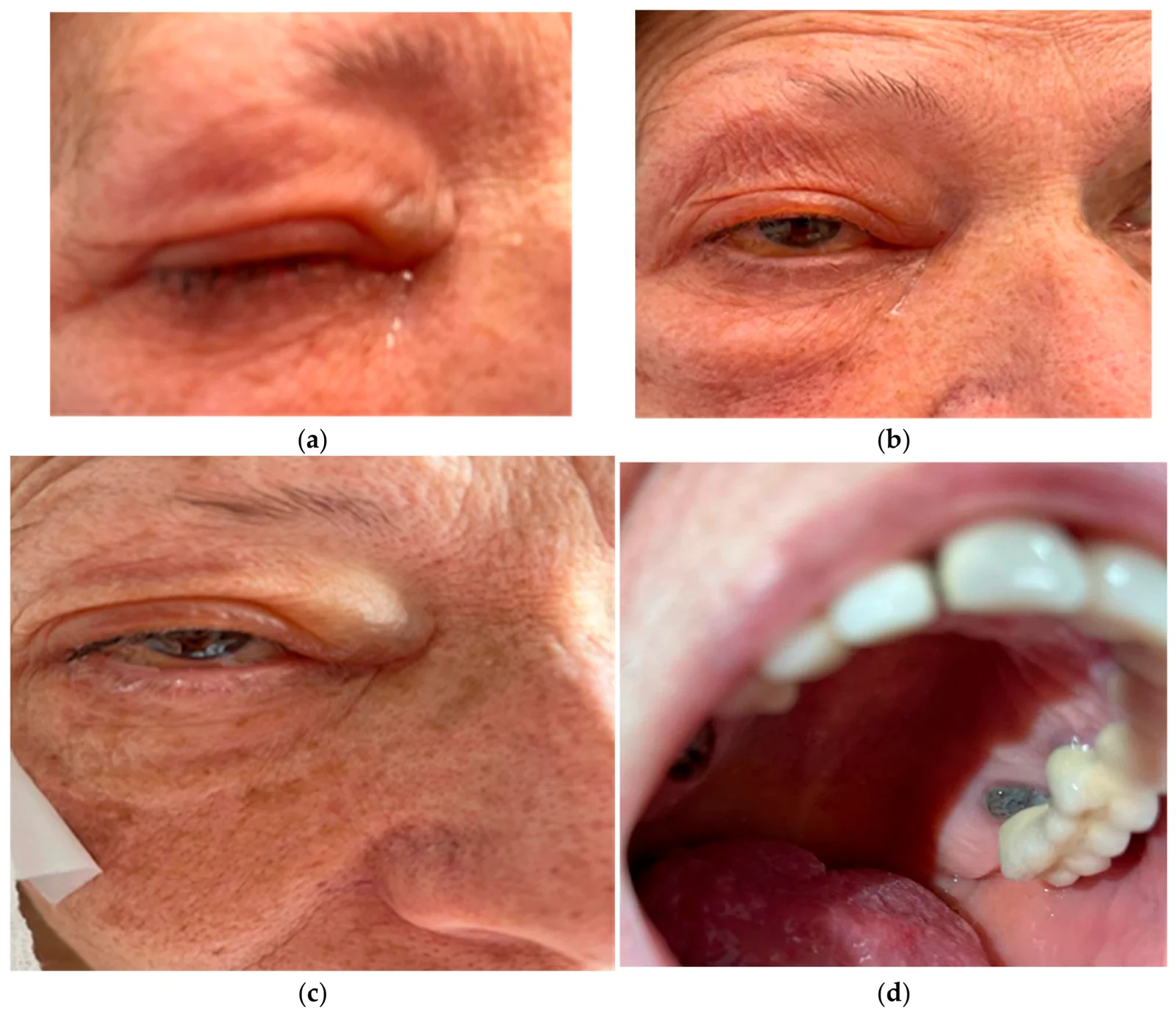
(**a**–**d**) Naso-orbital clinical appearance, exophthalmos, ptosis, and gingival lesion.

The symptoms did not respond to the administered analgesics and anti-inflammatory agents or to the ophthalmic drops used. After 13 h, at 21:00, the patient presented to the hospital, where she was evaluated simultaneously by a neurologist and a hematologist. Neurological examination ruled out intracranial pathology, raising the possibility of an orbital cause for the exophthalmos. Prior clinical experience with mucormycosis in patients with hematologic malignancies also raised suspicion of mucormycosis (Figure 5).

The differential diagnosis of right-sided exophthalmos (proptosis) included: (1) infections—bacterial (orbital cellulitis, abscess), fungal (Aspergillus, mucormycosis), parasitic (trichinellosis, echinococcosis), and Nocardia, which was considered a possible etiology in this patient; (2) inflammatory conditions—idiopathic orbital inflammatory syndrome (pseudotumor), Langerhans cell histiocytosis, sarcoidosis, and Graves’ ophthalmopathy; (3) tumors—capillary hemangioma, fibrous tumors, fibrosarcoma, optic nerve glioma, granulocytic sarcoma associated with leukemia, lymphomas, plasmacytomas, meningiomas, and metastases, particularly in the context of the patient’s history of cholangiocarcinoma; (4) trauma—orbital fractures and foreign bodies; and (5) other conditions—orbital varices, arteriovenous malformations, mucoceles, and dermoid and epidermoid cysts.

**Figure 6:**
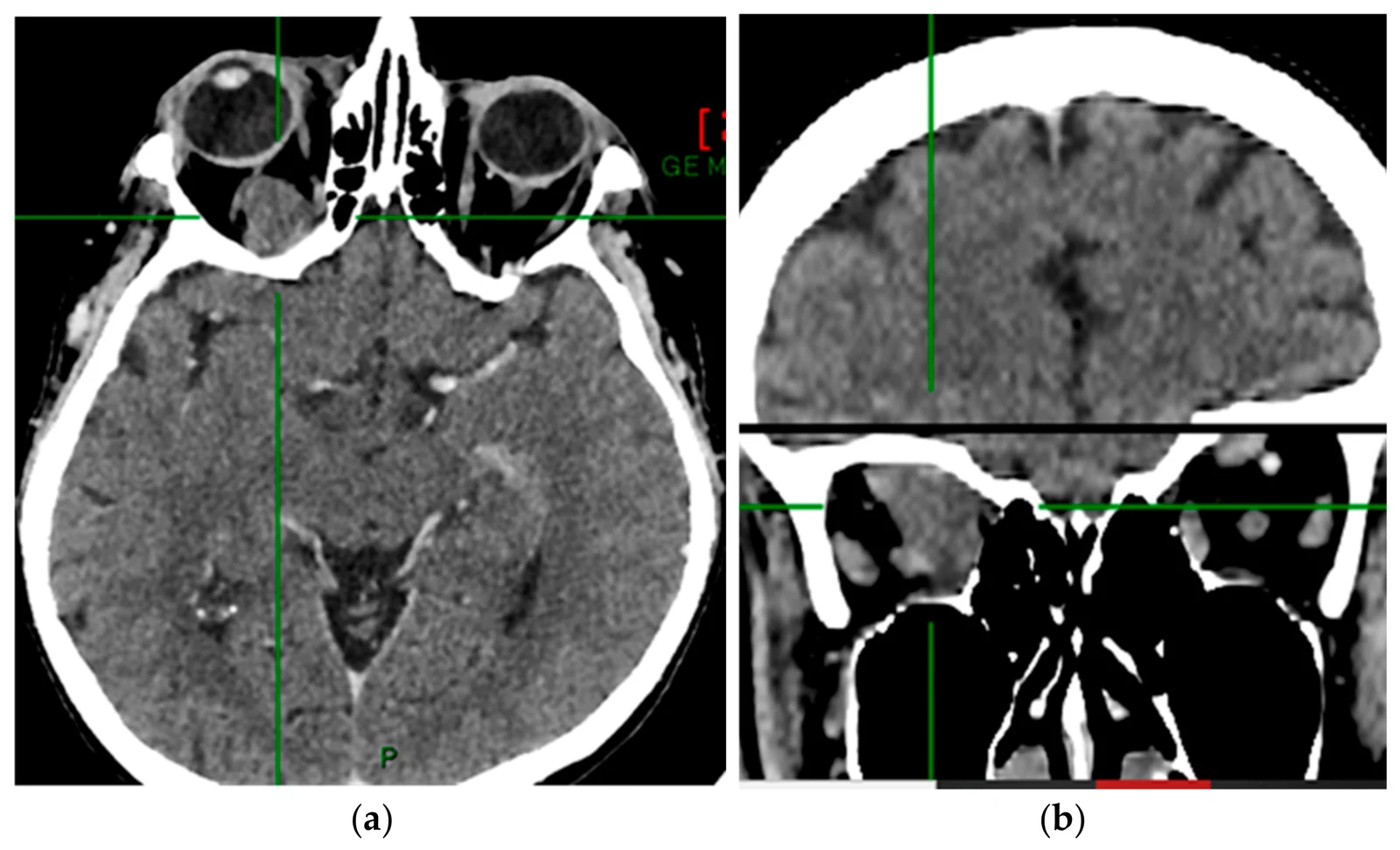

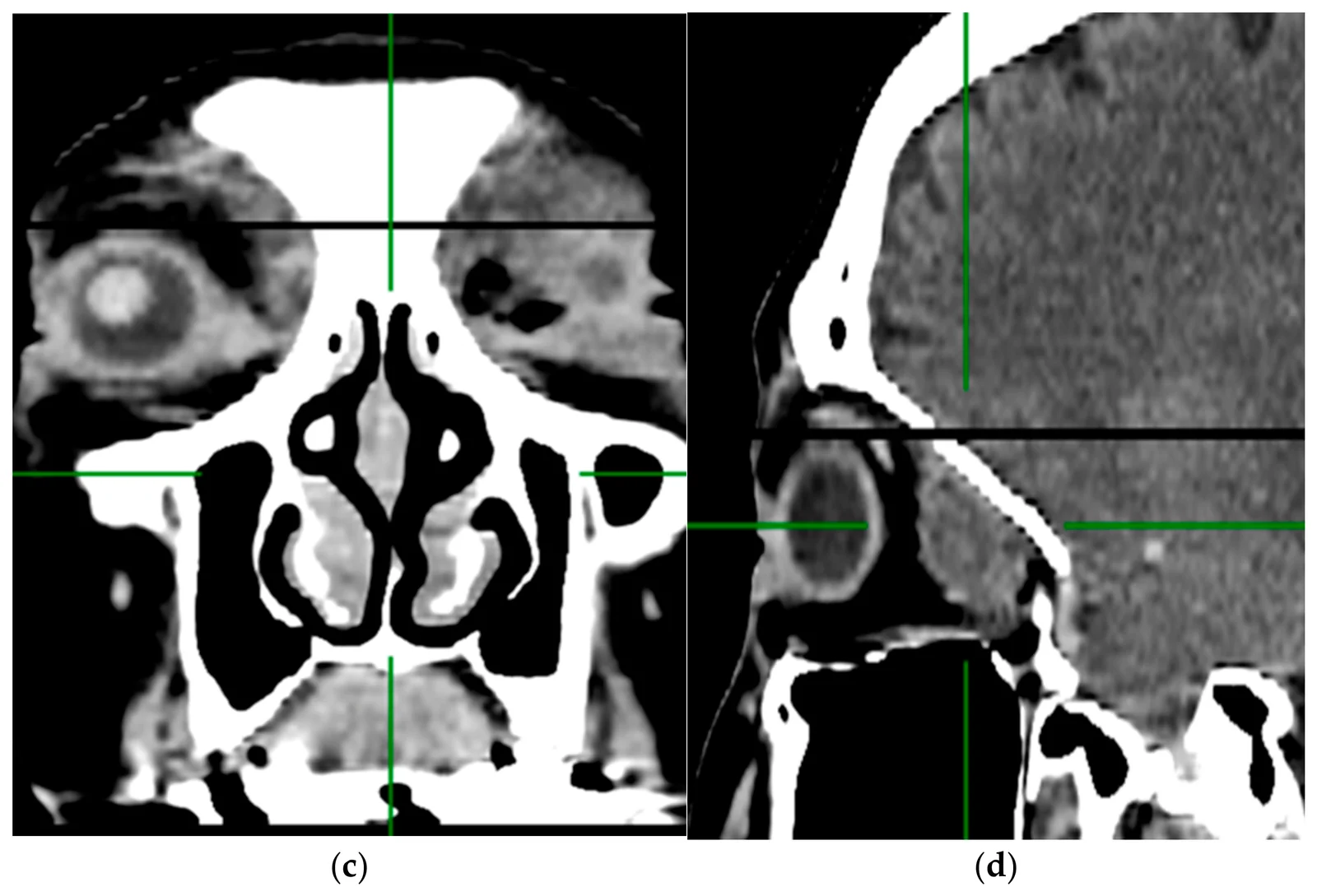
(**a**–**d**) Asymmetric right-sided exophthalmos caused by an intraorbital retroocular nodular lesion with irregular margins and heterogeneous contrast enhancement, in contact with the superior and inferior rectus muscles and the optic nerve; minimal mucosal thickening of the right nasal concha, maxillary sinus, and ethmoid sinus.

A brain CT scan was performed, revealing right-sided asymmetric exophthalmos caused by an intraorbital retroocular nodular lesion with irregular contours and isodense attenuation, in contact with the superior and inferior rectus muscles as well as the optic nerve, as well as minimal mucosal thickening of the right nasal concha, maxillary sinus, and ethmoid sinus (Figure 6).

**Figure 7:**
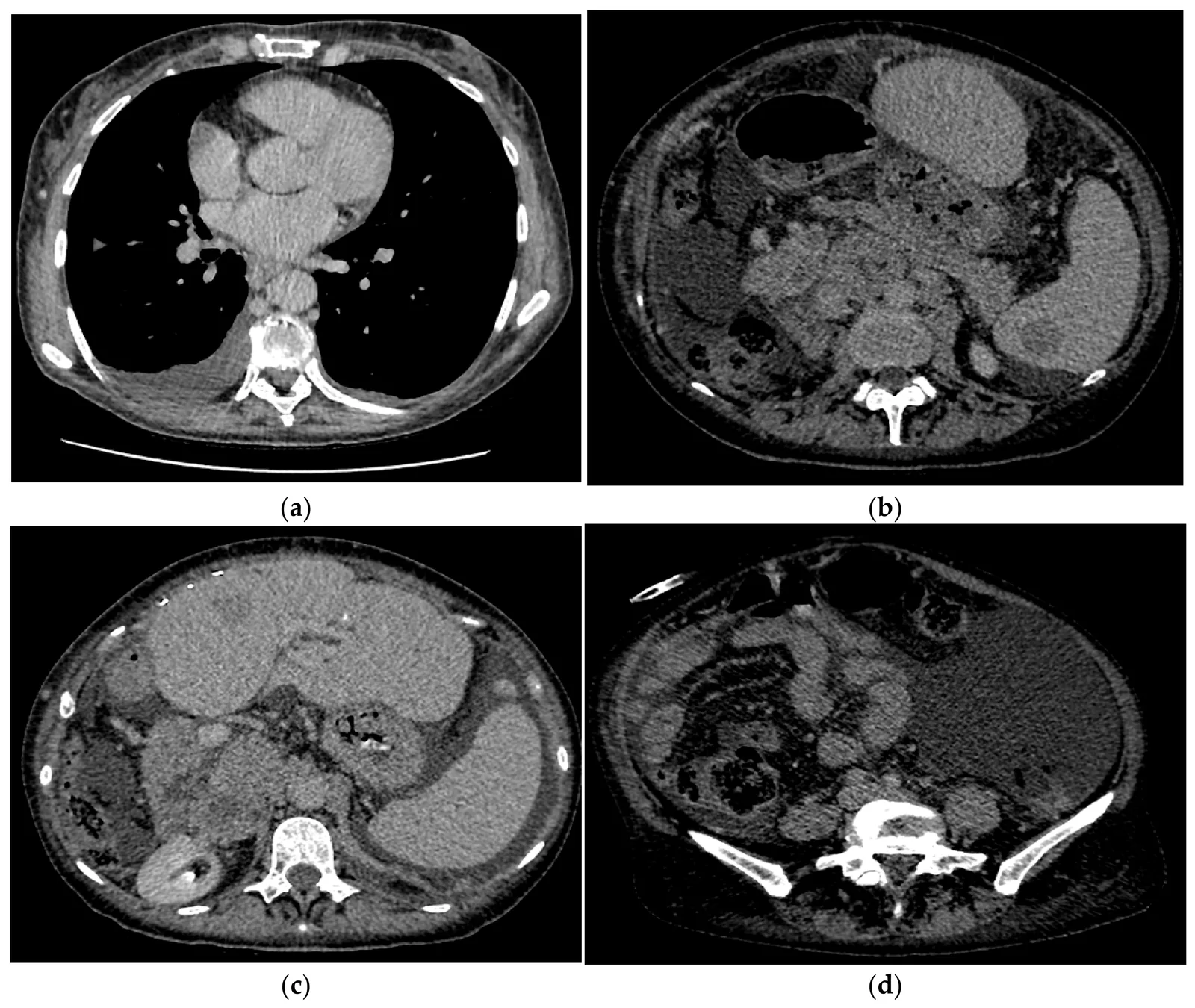
(**a**–**d**) CT of the thorax, abdomen, and pelvis (TAP CT).

CT of the thorax, abdomen, and pelvis (CT TAP) revealed a right pleural effusion and a very thin left pleural layer; the left hepatic lobe (LHL) remained heterogeneous, with some hypodense areas not corresponding to the February PET scan; retroperitoneal lymphadenopathy, also evident on the previous PET scan, without radiotracer uptake; a heterogeneous spleen with a nodular lesion, without PET correlation; and moderate ascites (Figure 7).

Laboratory findings were as follows: WBC = 6400/mm^3^; neutrophils = 5080/mm^3^; Hb = 8 g/dL; platelets = 138,000/mm^3^; CRP = 7.8 mg/L; TSH = 81.2 mU/L (normal range 0.5–4.7), attributed to nivolumab treatment; CA125 = 127 U/mL (normal range 0–30), in the context of ascites; D-dimer = 2279 ng/mL (normal range 45–500). Evidence of cholestasis and hepatocytolysis was observed: alkaline phosphatase (ALP) = 812 U/L; GGT = 525 U/L; AST (TGO) = 179 U/L; ALT (TGP) = 95 U/L. Multidrug-resistant *Klebsiella pneumoniae* was isolated from sputum.

**Figure 8:**
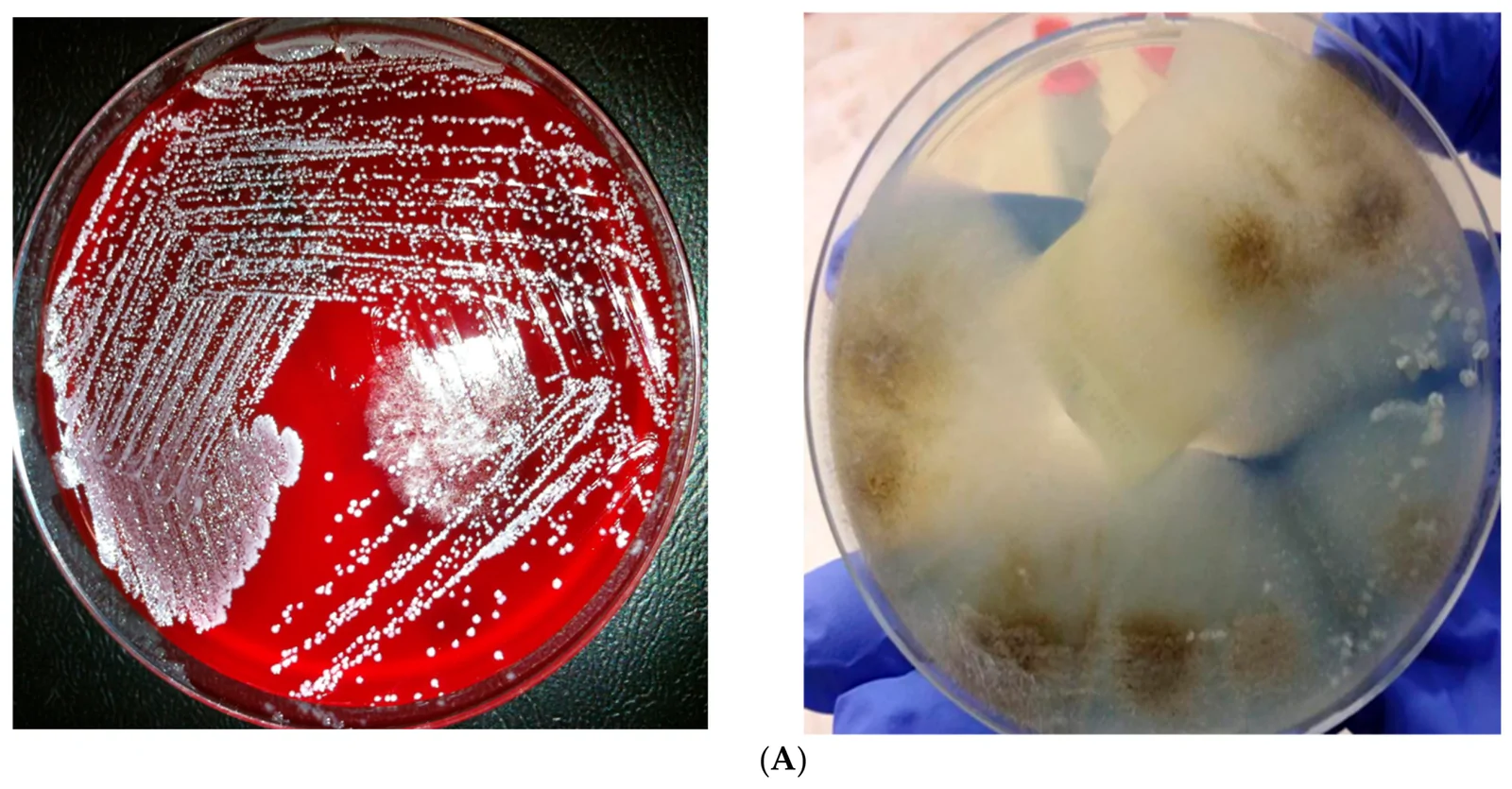

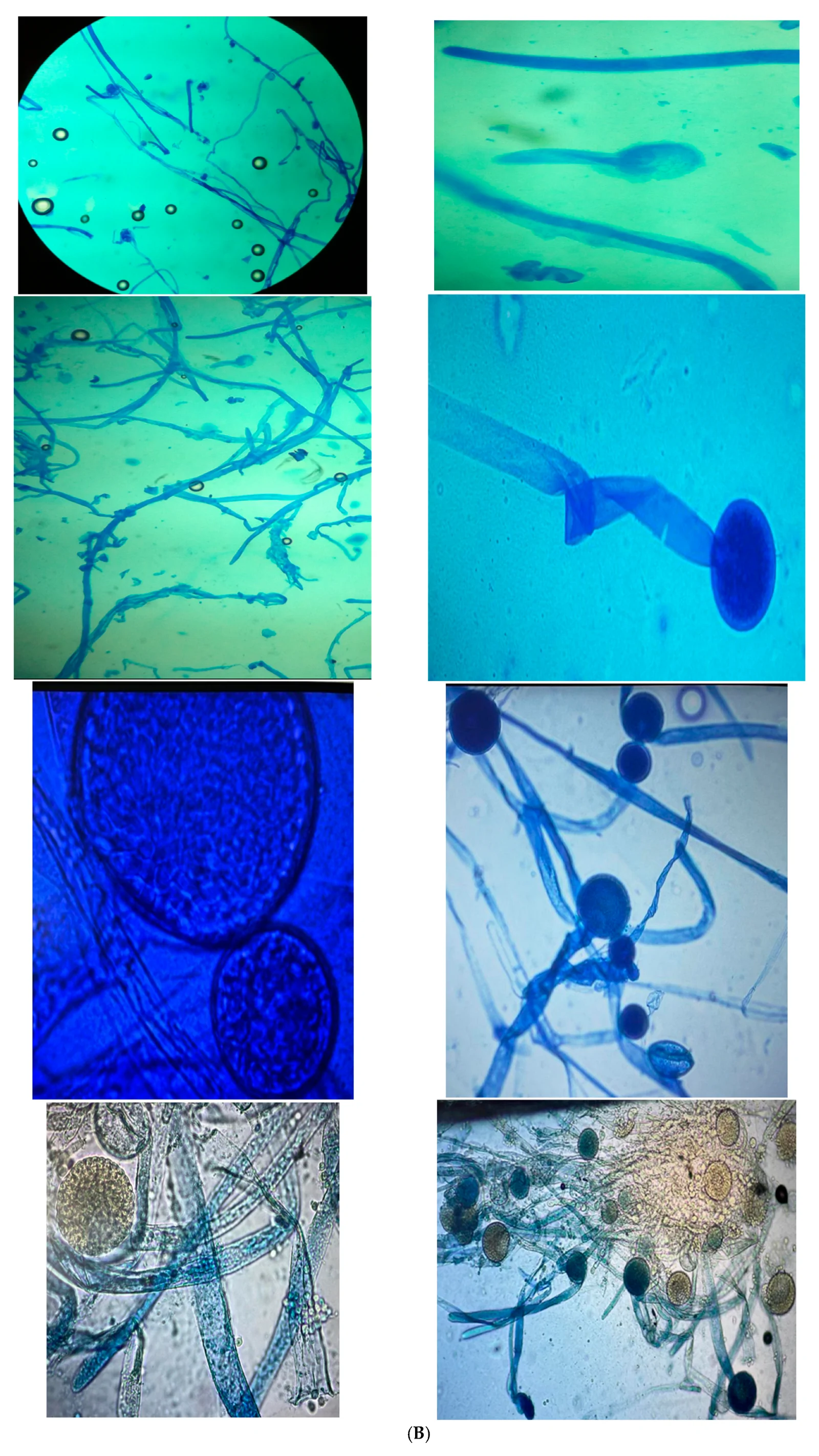

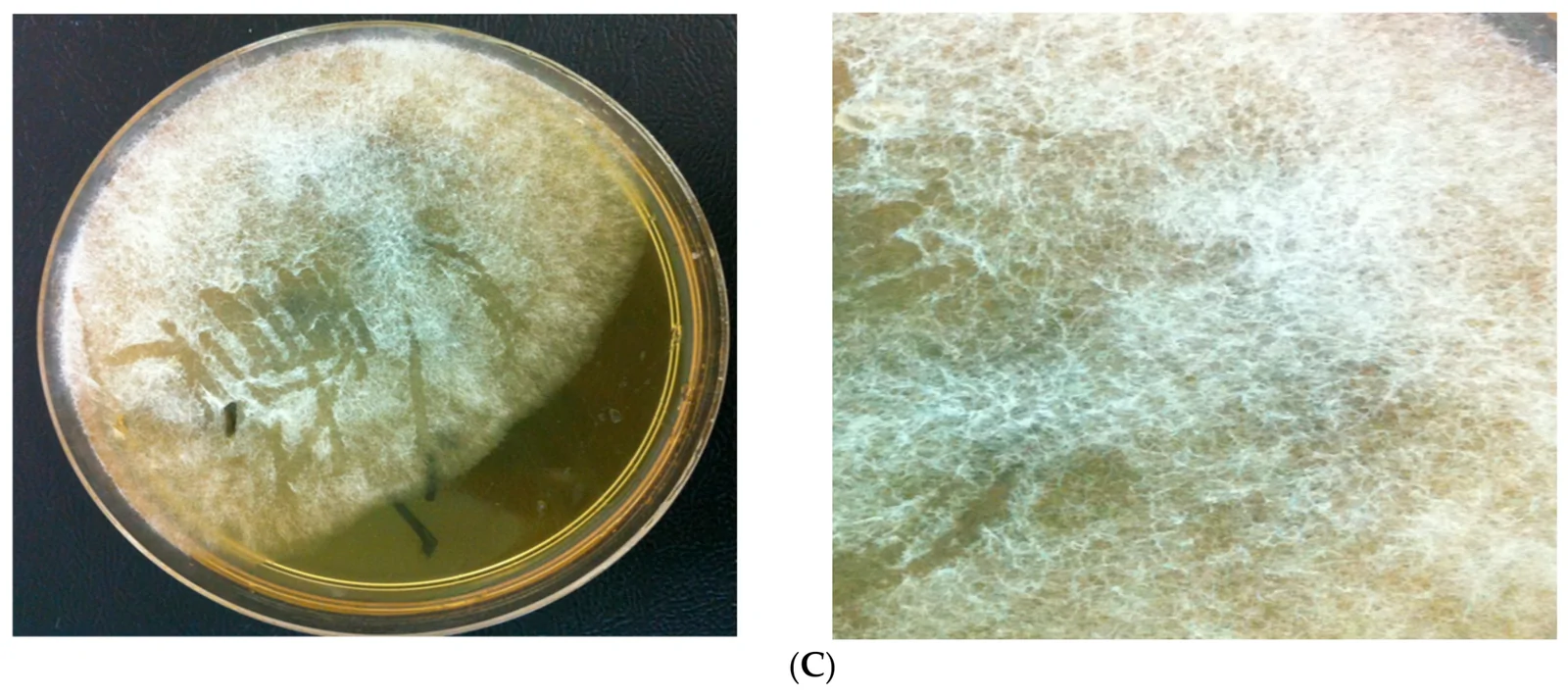
(**A**) Mold growth observed on blood agar culture medium. (**B**) Direct microscopic examination using a lactophenol-stained preparation revealed mycelium composed of unicellular, non-septate, branched hyphae; vertical branches, including sporangiophores bearing sporangia, arising from the mycelium—consistent with organisms of the order *Zygomycetales*, family *Mucoraceae* (*Mucor* species). The hyphae were oriented at approximately 90 degrees, club-shaped, with a bulbous enlargement at one end. The micrographs were captured under 100× magnification. (**C**) Culture on Sabouraud dextrose agar (SDA), sheep blood agar (SBA), and cystine–lactose–electrolyte-deficient (CLED) agar revealed abundant (++++) filamentous fungal growth within 48 h; the cottony, woolly appearance was suggestive of infection with zygomycetes.

Cultures collected from the right nasal cavity and the right conjunctival sac revealed the presence of fungi (Figure 8A–C).

Treatment consisted of prophylactic isolation measures, protection of healthcare personnel with appropriate equipment to prevent transmission to other immunocompromised patients, and frequent ventilation. Amphotericin B was not available at that time; therefore, treatment was initiated with isavuconazole at a dose of 600 mg/day for 2 days, followed by 400 mg/day for 2 weeks, and subsequently 200 mg/day for 6 months.

Dexamethasone was administered for 3 days for its analgesic and anti-inflammatory effects but was subsequently discontinued due to a high suspicion of mucormycosis. Treatment was continued with osmotic and loop diuretics—mannitol, furosemide, and spironolactone—as well as analgesics, albumin, hepatoprotective agents, and propranolol for the associated liver disease. The *Klebsiella pneumoniae* infection was controlled with third-generation cephalosporins. For established hypothyroidism, attributed to nivolumab therapy, the patient received levothyroxine (Euthyrox) to control thyroid dysfunction. Given the known angioinvasive, microembolic, and necrotic nature of mucormycosis, therapeutic-dose anticoagulation was also administered.

There was also the possibility of orbital metastasis from cholangiocarcinoma concurrent with rhinosinusal mucormycosis. A biopsy was conducted to clarify this was not feasible in the presence of an invasive fungal infection. It was decided to treat the fungal infection first, with biopsy to be performed if the retroorbital tumor persisted.

The clinical course was remarkable: diplopia and pain resolved within 24 h; eyelid edema resolved within 5 days; ocular motility recovered after 5 days; eyelid ptosis was completely corrected within 10 days; exophthalmos resolved within 2 weeks; ascites and peripheral edema resolved within 14 days; and cultures became negative within 10 days.

**Figure 9:**
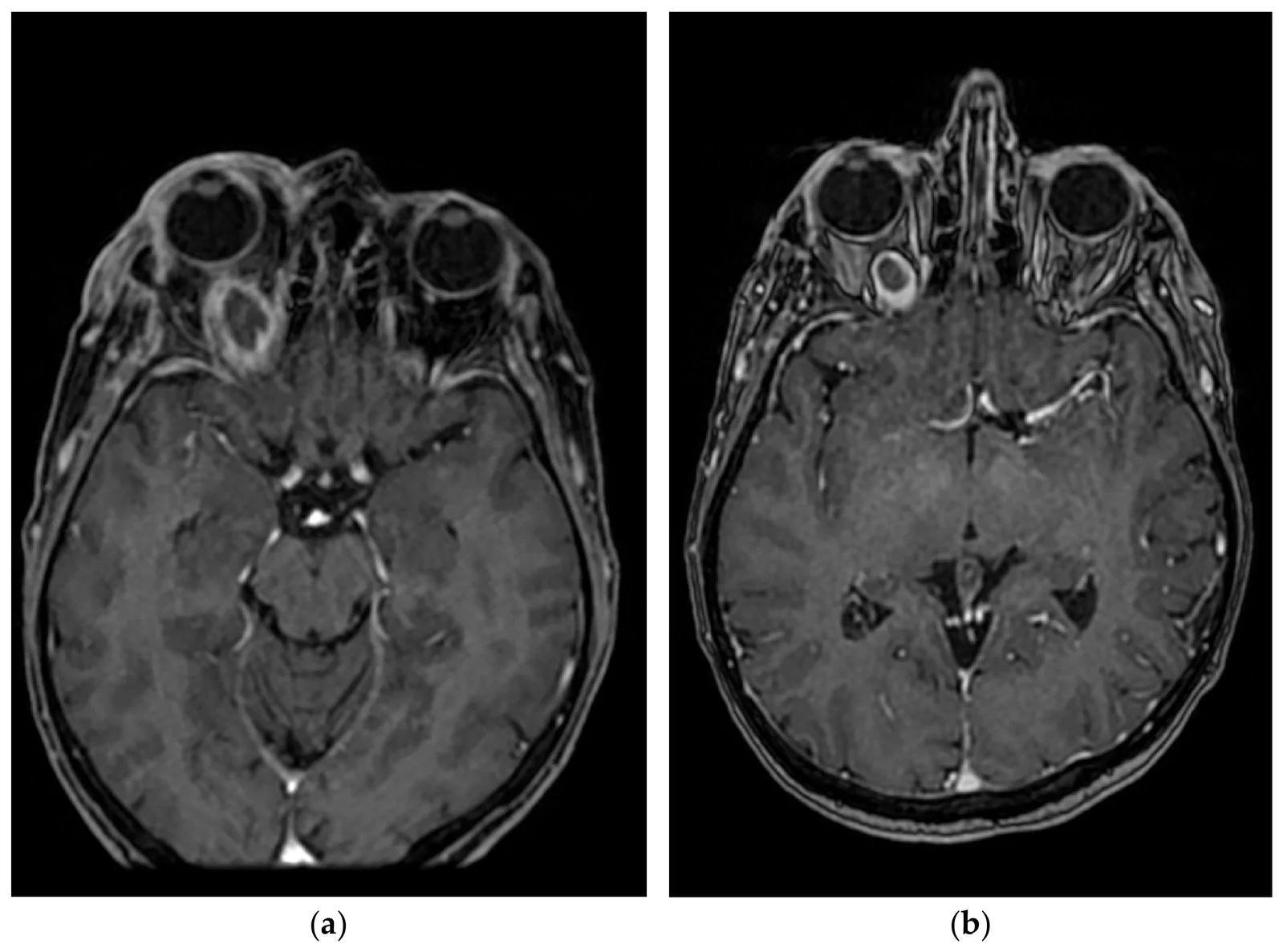

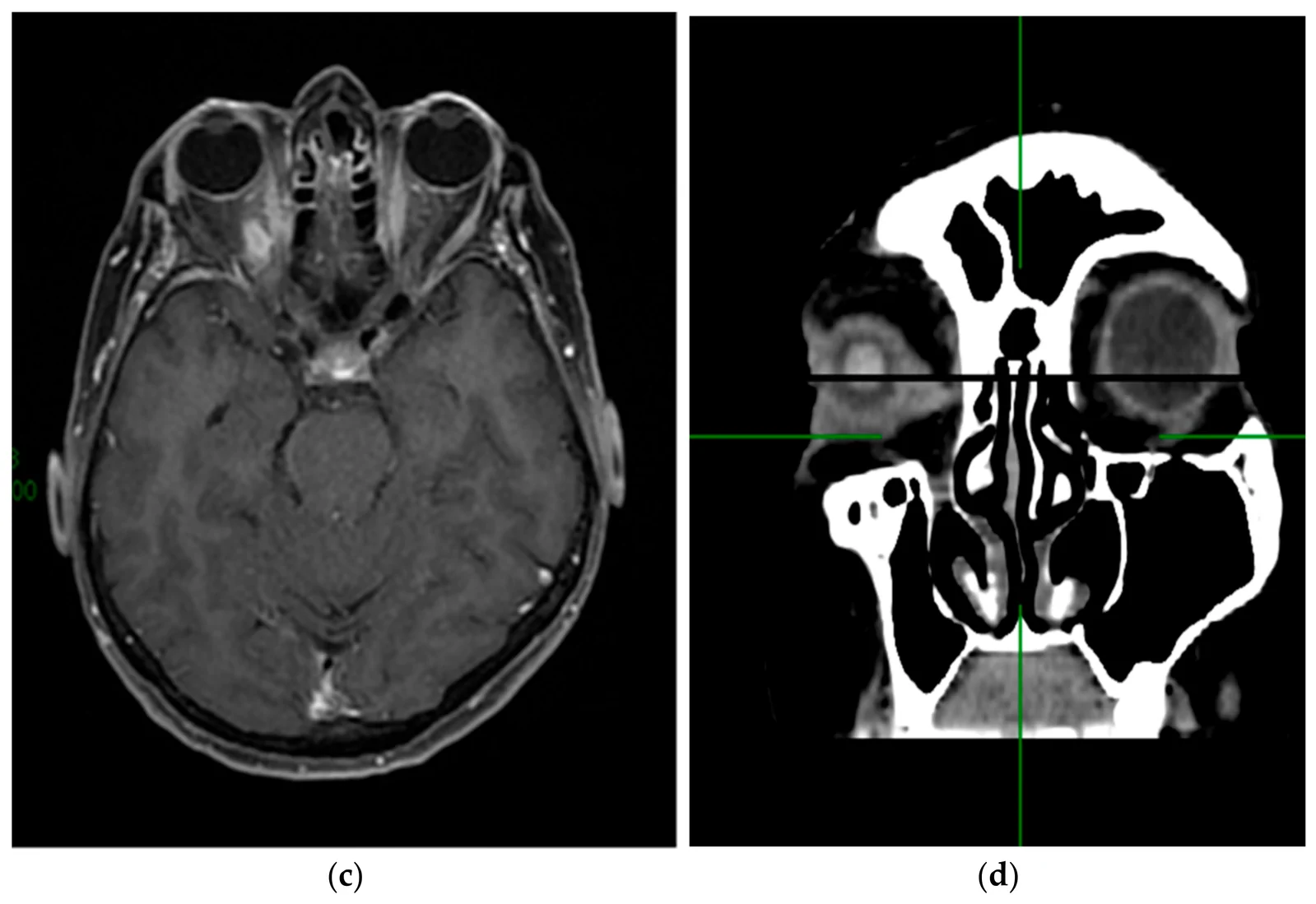
Imaging progression: (**a**) 26 June 2023, at diagnosis; (**b**) 13 July 2023—2 weeks later; (**c**) 31 August 2023—2 months later; (**d**) normalization of the nasal and sinus mucosa. Near-complete remission of the orbital lesion was observed after 3 months.

Imaging findings demonstrated a significant reduction in the orbital mass at 15 days, with near-complete regression at 2 and 3 months after symptom onset. The mucosal thickening of the nasal turbinate, as well as that of the maxillary and ethmoid sinuses, resolved. At that time, the suspicion of concomitant orbital metastasis from cholangiocarcinoma was ruled out (Figure 9).

After 6 months, with the fungal infection in remission (clinically, biologically, microbiologically, and on imaging) and following normalization of liver function, monthly nivolumab infusions were resumed to prevent recurrence or progression of cholangiocarcinoma. The patient’s presentation within 13 h of symptom onset, clinical suspicion, prompt imaging prior to microbiological confirmation, and interdisciplinary collaboration were the cornerstones of therapeutic success in this case, with survival of 16 years from the onset of cholangiocarcinoma, 7 years from recurrence, and 2 year following the mucormycosis infection.

Death occurred 2 years later, on 30 June 2025, due to disease progression with multiple abdominal metastases, subocclusive syndrome secondary to peritoneal carcinomatosis, *Escherichia coli* sepsis of gastrointestinal origin in the context of intestinal obstruction, gastrointestinal hemorrhage due to esophageal variceal rupture, hypotension, and respiratory failure.

## 3. Case Series

The experience of the Hematology Clinic in Sibiu includes eight cases of mucormycosis: five cases of rhinosino-orbito-cerebral mucormycosis (2013, 2021, 2023, 2024), one case of pulmonary mucormycosis (2024), two cases of cutaneous mucormycosis (2021, 2024), and one case of urinary mucormycosis. These anatomical categories were not mutually exclusive, as Case 7 initially presented with cutaneous mucormycosis and subsequently progressed to rhino-sino-orbito-cerebral involvement.

**Figure 10:**
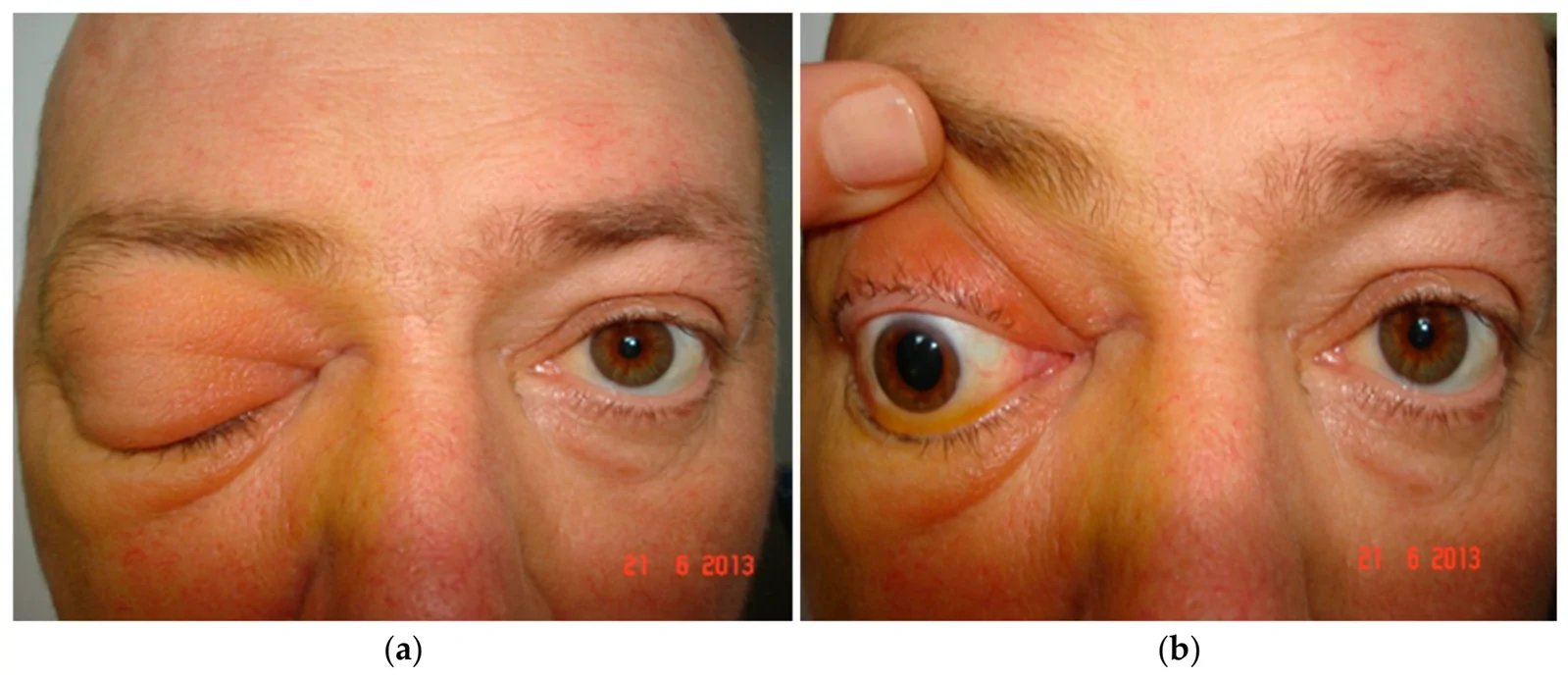
(**a**,**b**) Clinical evolution of the rhino-orbito-sinusal lesion in Case No. 2.

**Figure 11:**
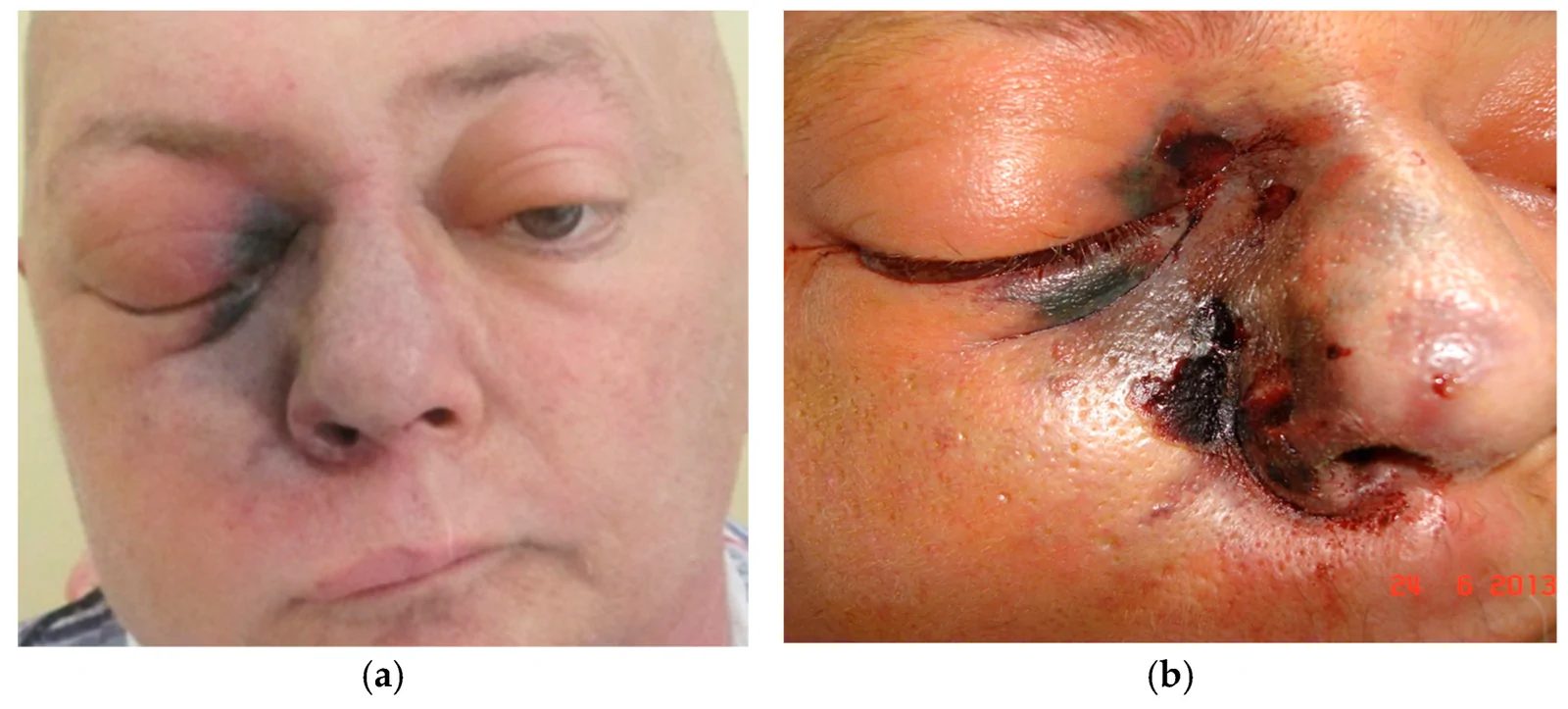
(**a**,**b**) Clinical evolution of the rhino-orbito-sinusal lesion in Case No. 2.

**Figure 12:**
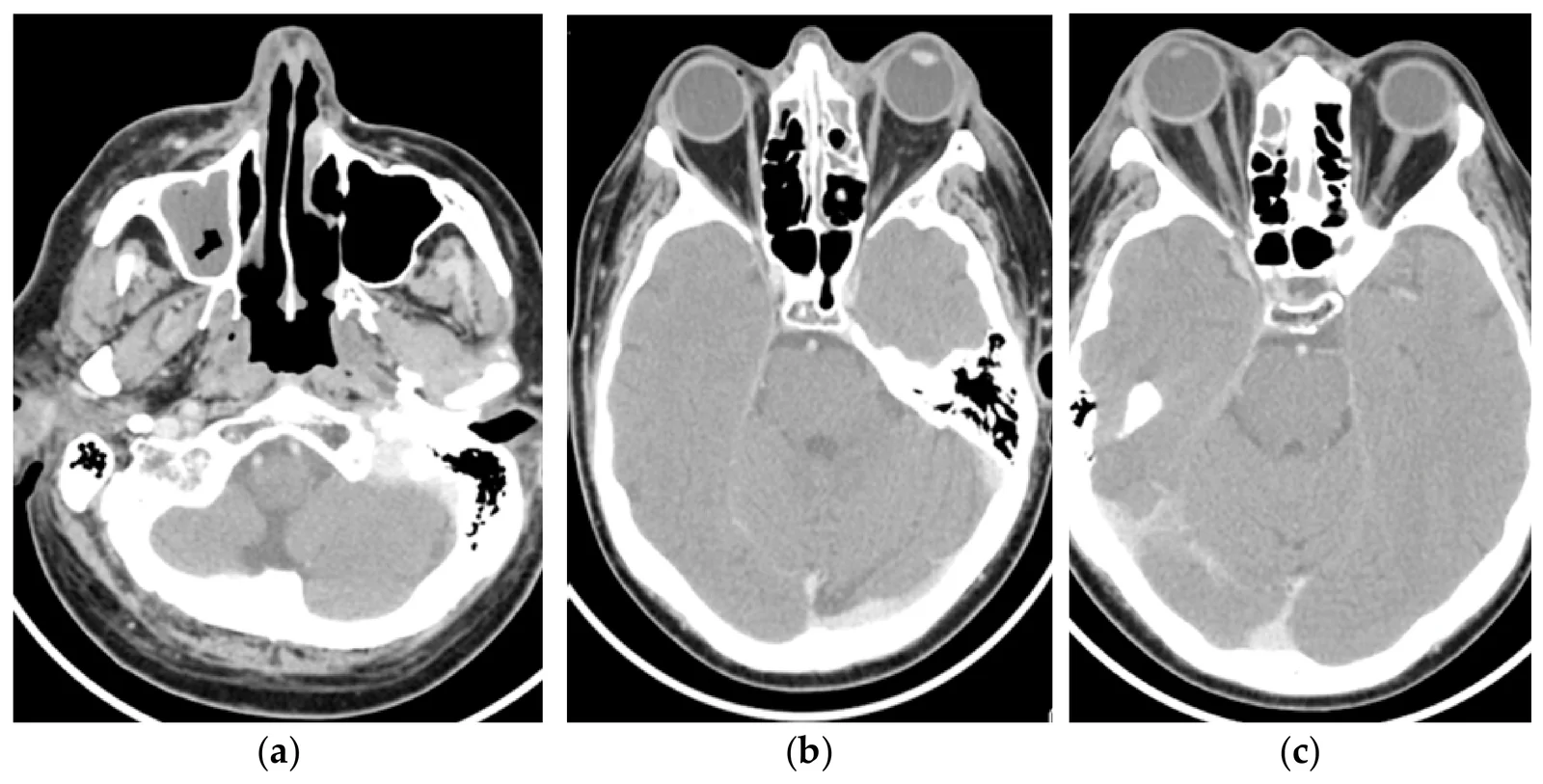
(**a**–**c**) Brain CT showing no evidence of intracranial pathology; right-sided exophthalmos with diffuse thickening of the retrobulbar fat, as well as thickening of the right eyelid and cheek, and right maxillary–ethmoid sinusitis.

**Figure 13:**
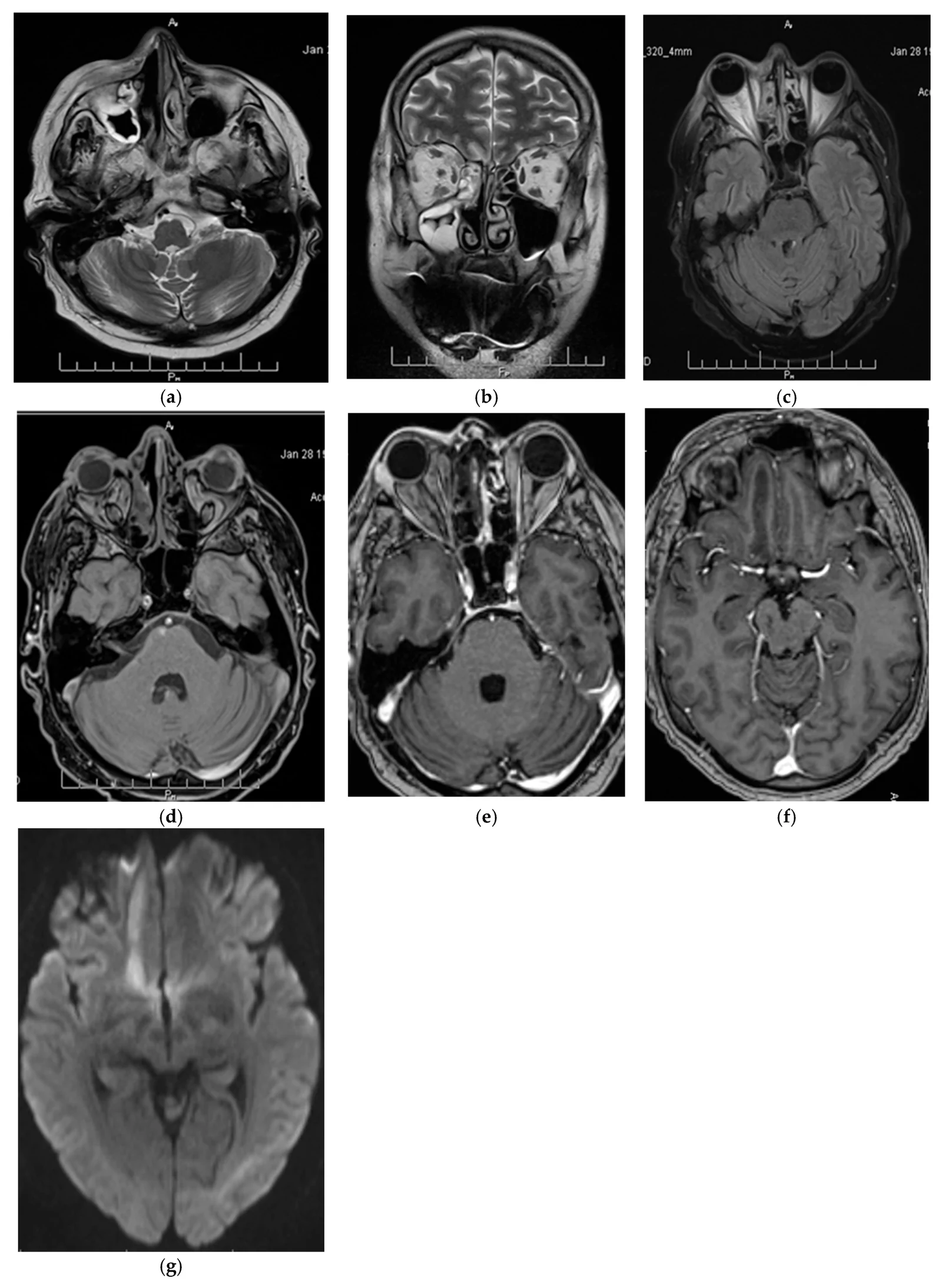
(**a**–**g**) Brain MRI showing periorbital gadolinium-enhancing infiltration of the right retroorbital fat, maxilloethmoidal sinusitis, and cellulitis involving the right eyelid, cheek, and infratemporal region. The right optic nerve demonstrated diffusion restriction and prechiasmatic gadolinium enhancement. Areas of diffusion restriction were also observed in the right frontobasal and left occipital regions (suggestive of acute ischemia in an embolic context or areas of cerebritis), along with small periventricular demyelinating lesions.

**Figure 14:**
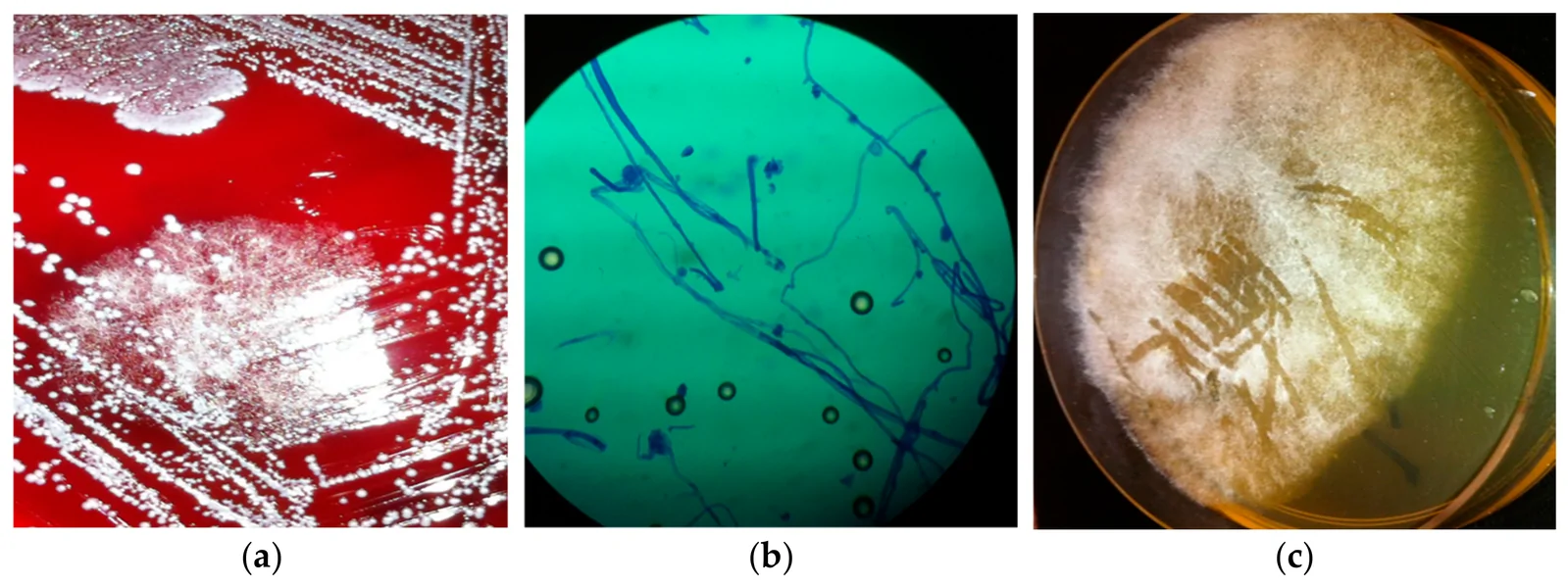
Mucormycosis: (**a**) Mold growth on blood agar culture medium; (**b**) direct microscopic examination using a lactophenol preparation. The micrographs were captured under 100× magnification; (**c**) culture on Sabouraud dextrose agar.

**Figure 15:**
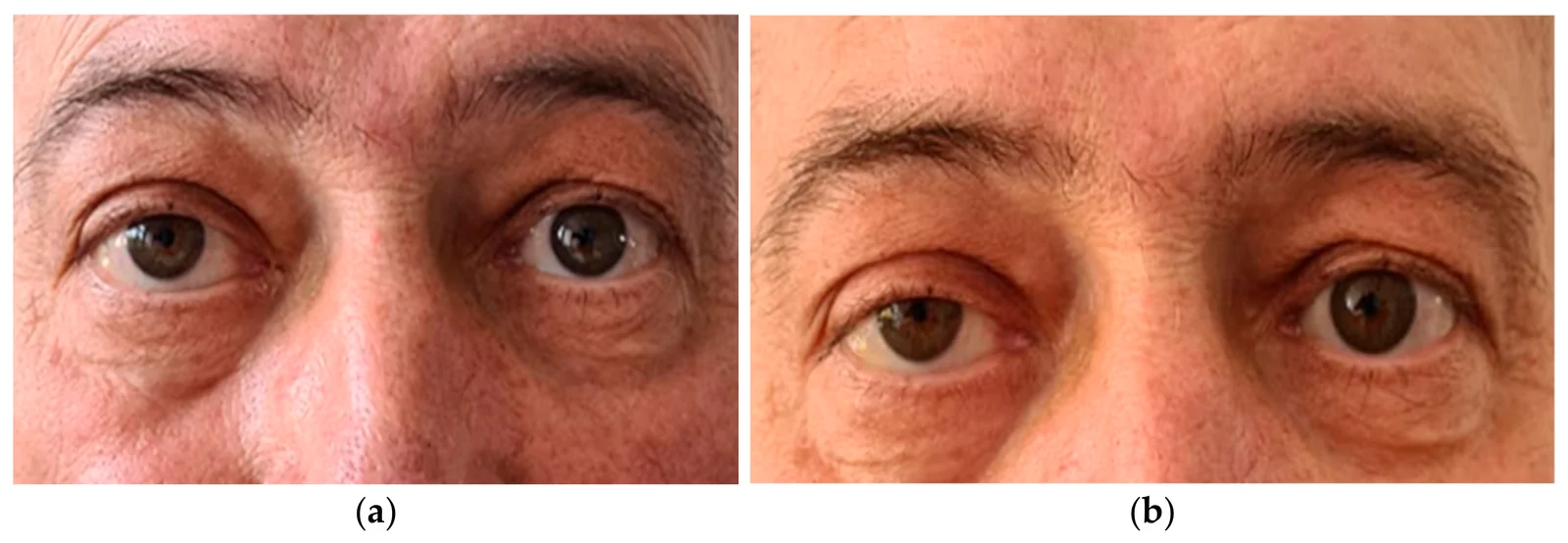
(**a**,**b**) Clinical appearance of rhinosino-orbital mucormycosis in case no. 3.

**Figure 16:**
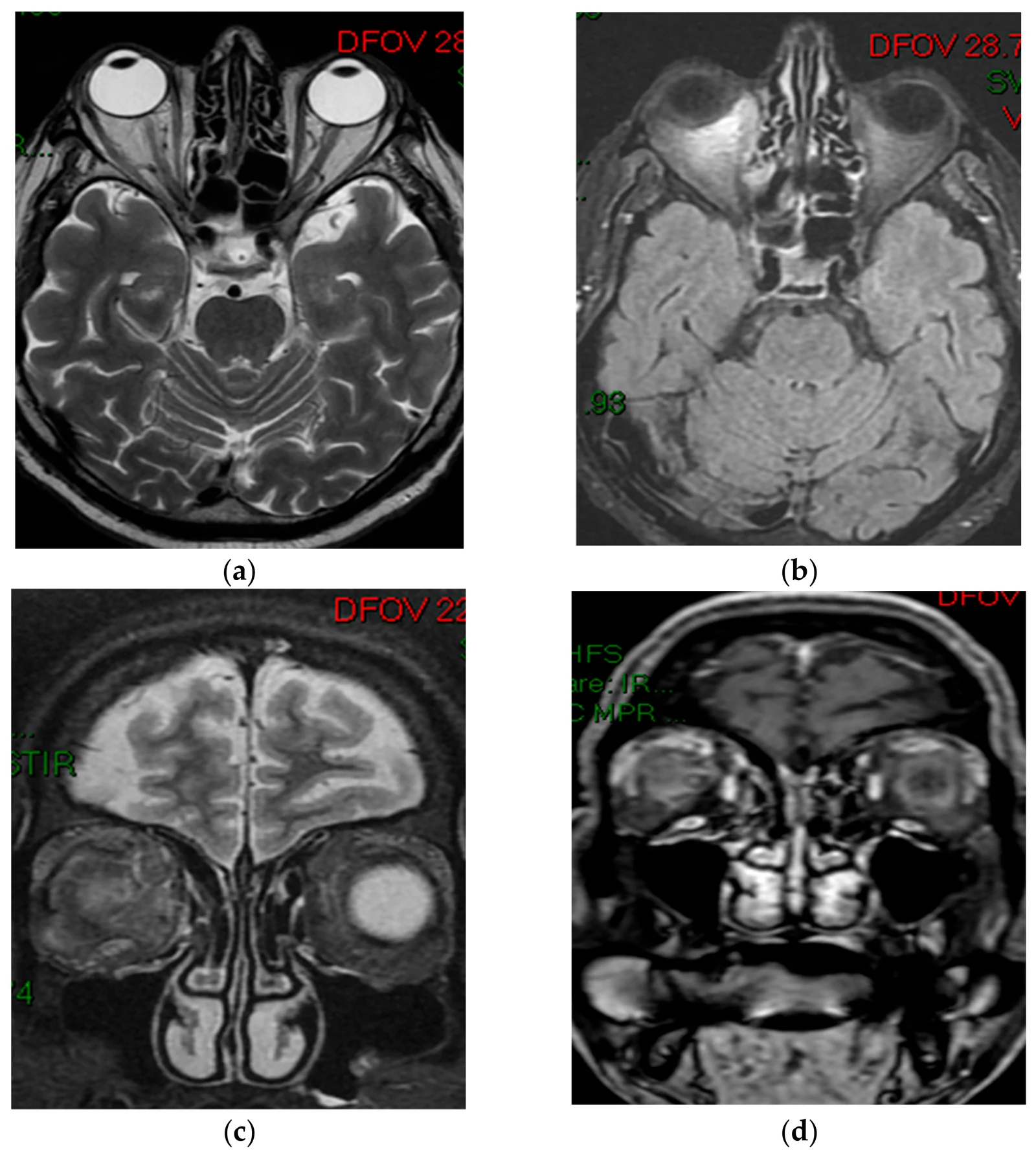
(**a**–**d**) Brain MRI in case no. 3.

**Figure 17:**
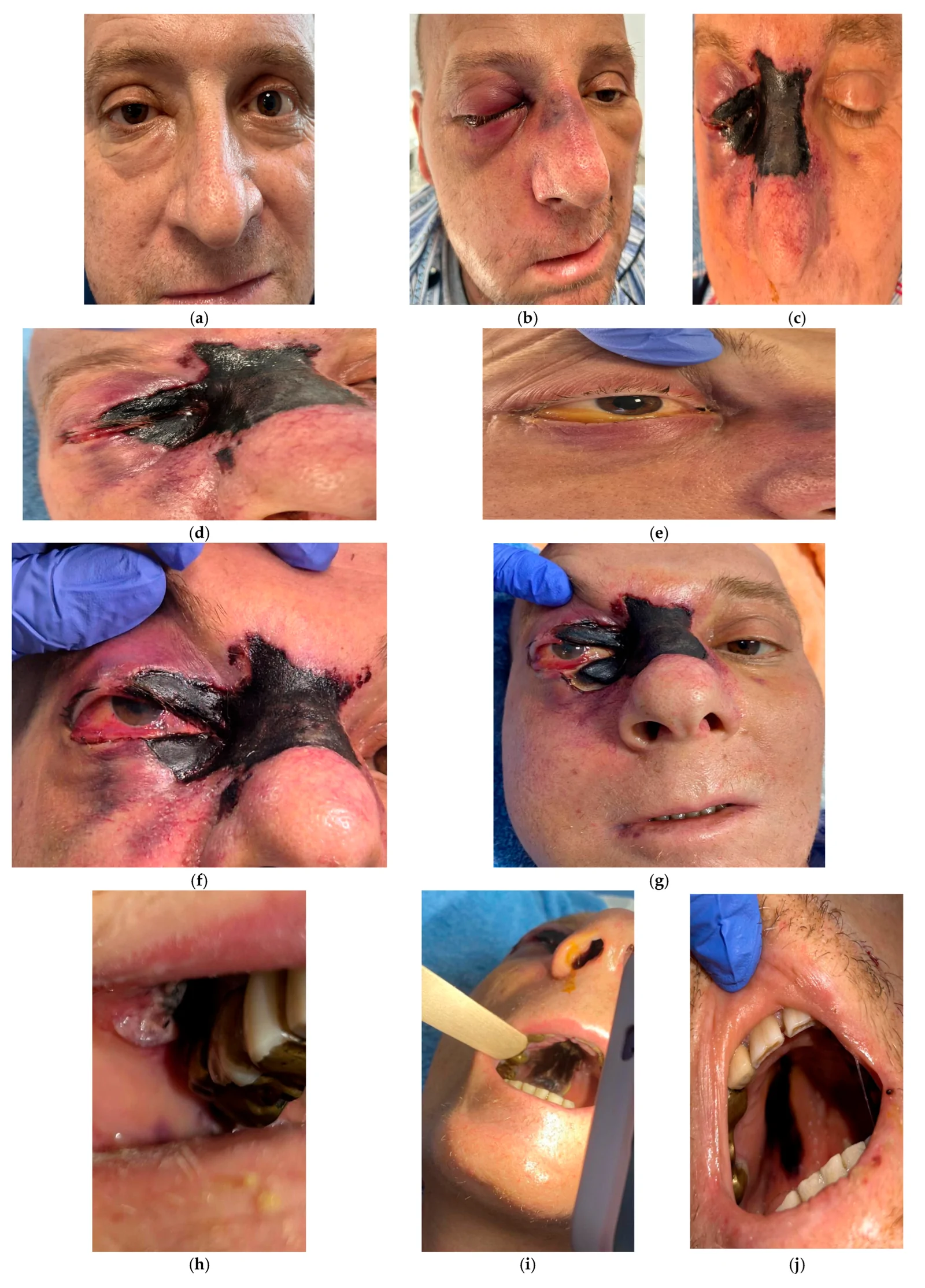

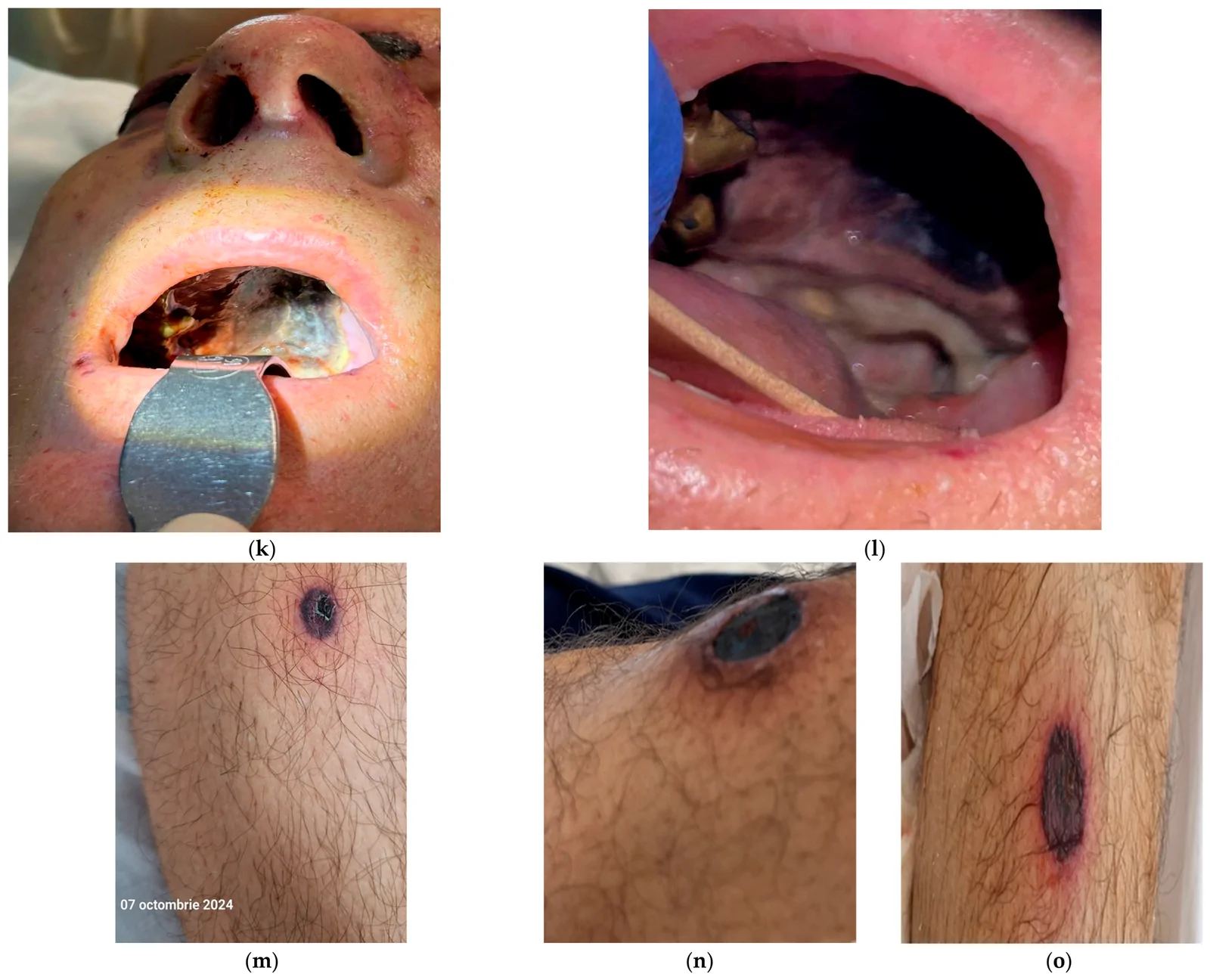
(**a**–**o**) Evolution of facio-orbital and oral lesions, as well as the solitary cutaneous lesion, in case no. 7.

**Figure 18:**
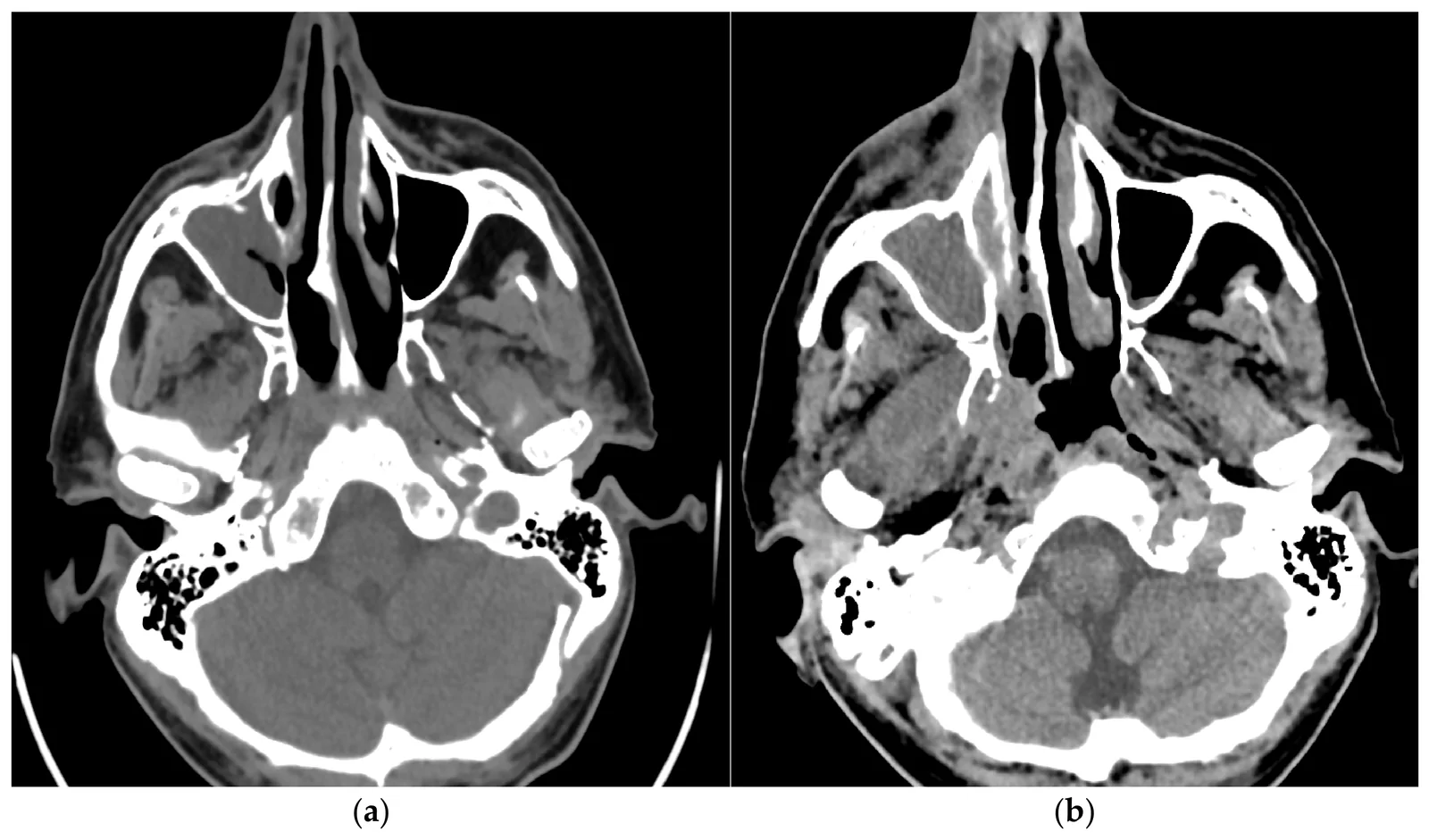
(**a**,**b**) Orbital CT scan (21 October 2024) showing no acute intracranial or orbital pathology, mild right frontal, maxillary, sphenoid, and ethmoid sinusitis, and right-sided proptosis in case no. 7.

**Figure 19:**
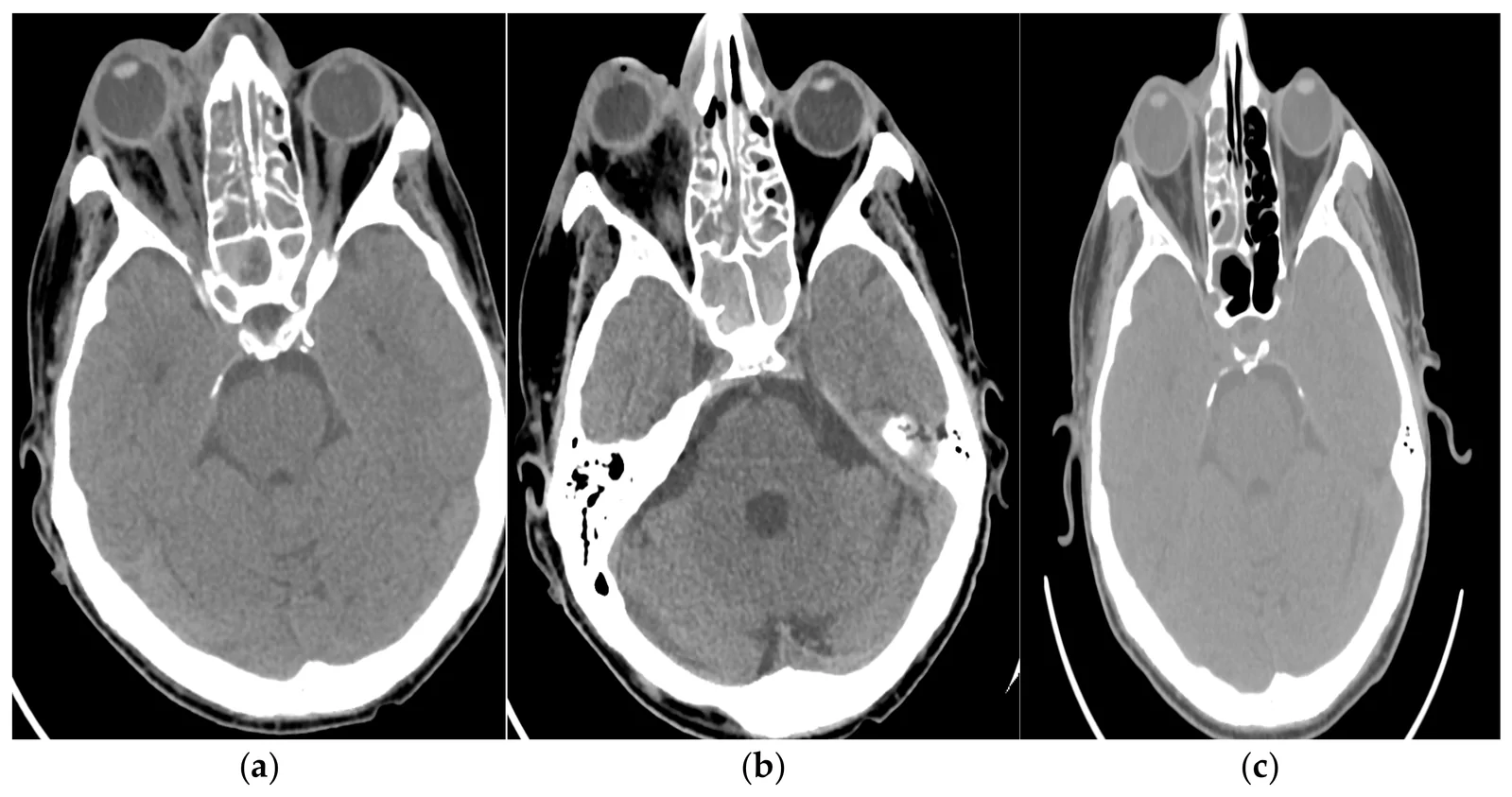
(**a**–**c**) Non-contrast cranial CT scan (3 December 2024) showing no acute intracranial lesions, with mucosal thickening and an isodense collection in the right maxillary sinus, right ethmoid air cells, and sphenoid sinus in case no. 7.

**Figure 20:**
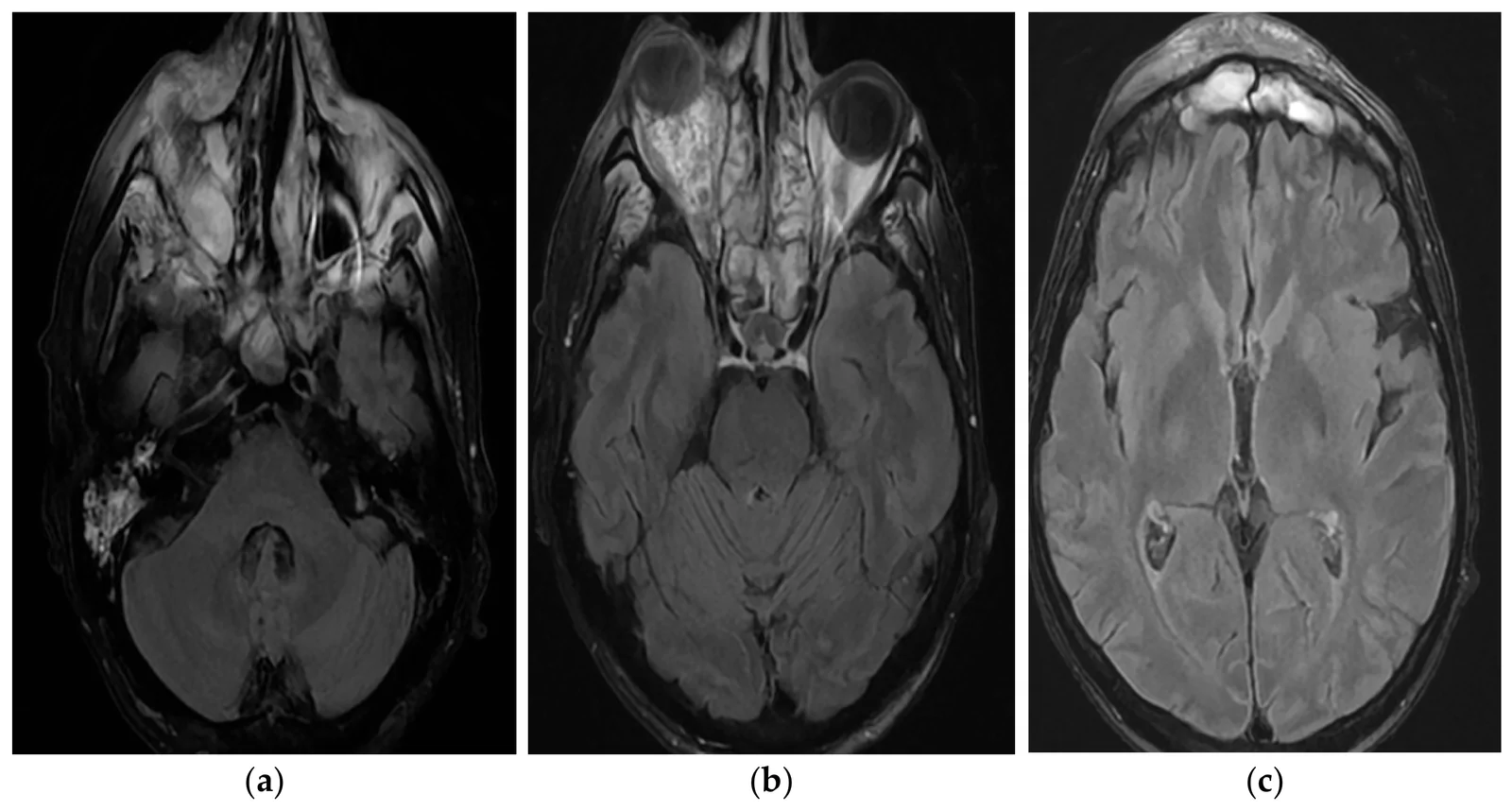
(**a**–**c**) Brain MRI (3 December 2024) demonstrating extension of the inflammatory process from the right sinus cavities, with involvement of the left ethmoid air cells and left frontal sinus, as well as marked progression of intraorbital inflammatory changes in case no. 7.

**Figure 21:**
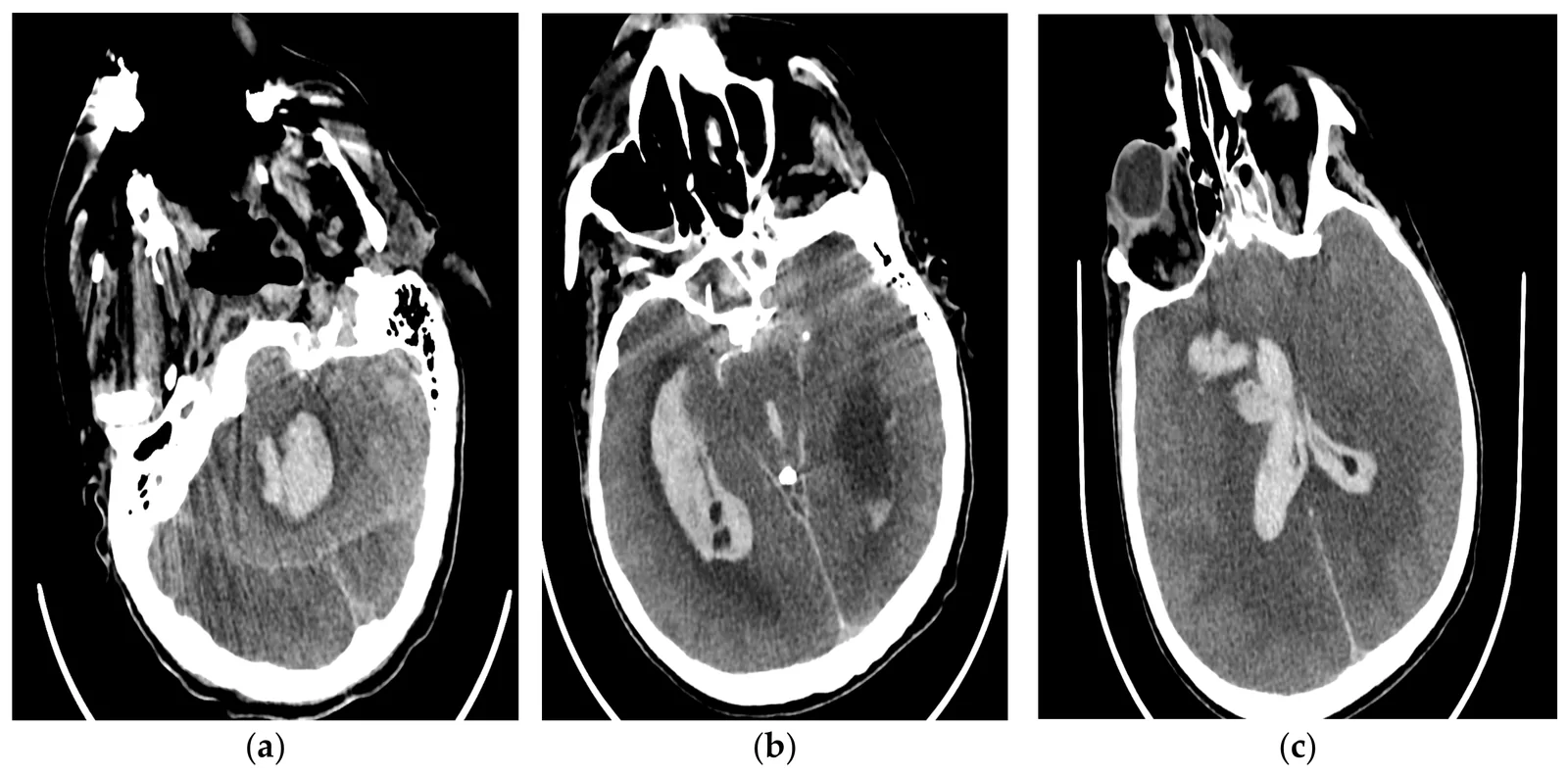
(**a**–**c**) Non-contrast cranial CT (19 December 2024) showing an intraparenchymal hemorrhagic lesion at the level of the right lentiform nucleus, associated with panventricular hemorrhage, subarachnoid hemorrhage, and diffuse cerebral edema with herniation at the level of the foramen magnum in case no. 7.

**Figure 22:**
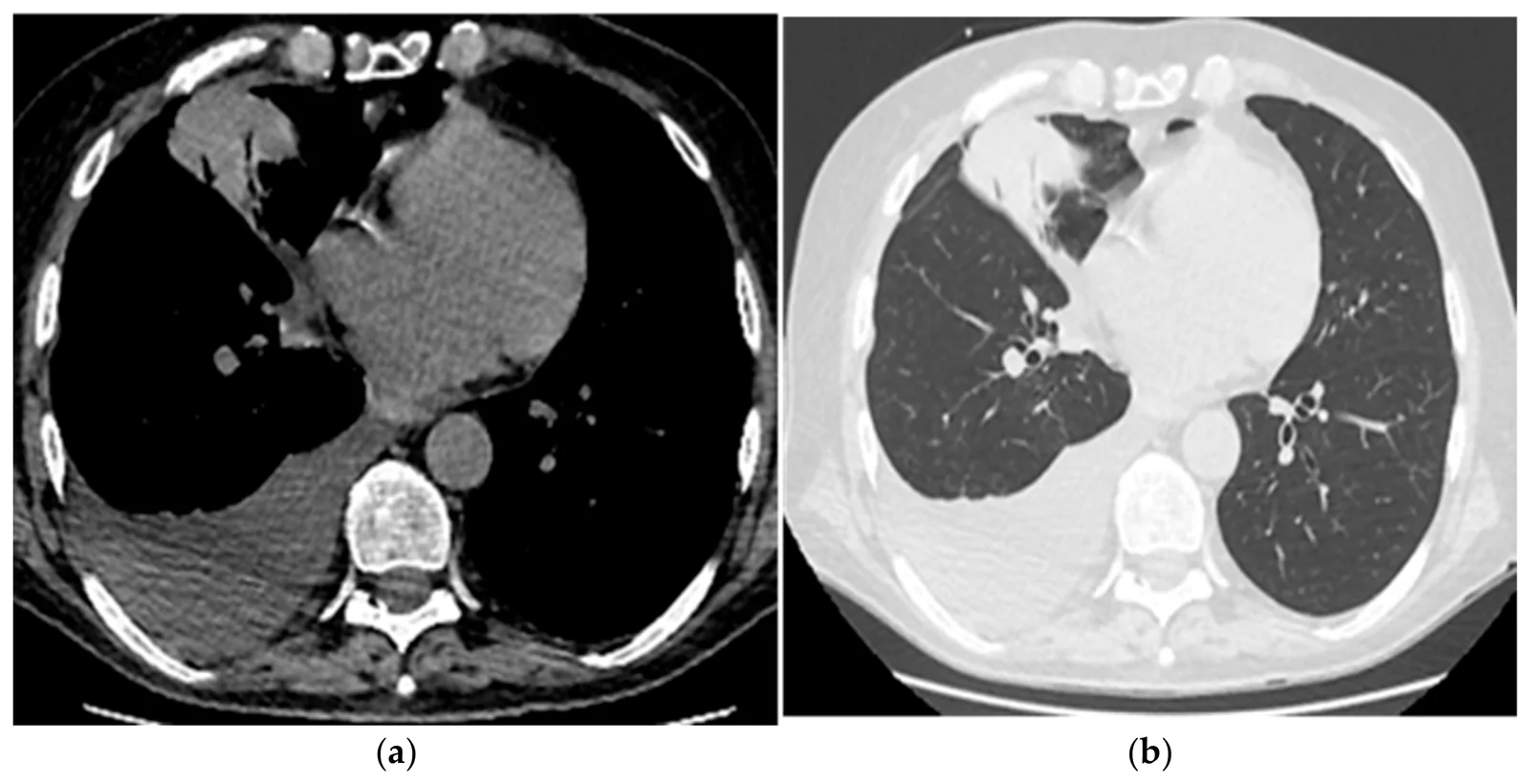
(**a**,**b**) CT of the thorax, abdomen, and pelvis (CT TAP) revealing pulmonary consolidation in the mediastinal and pulmonary regions, with air bronchograms and moderate pleural effusion in case no. 5.

**Figure 23:**
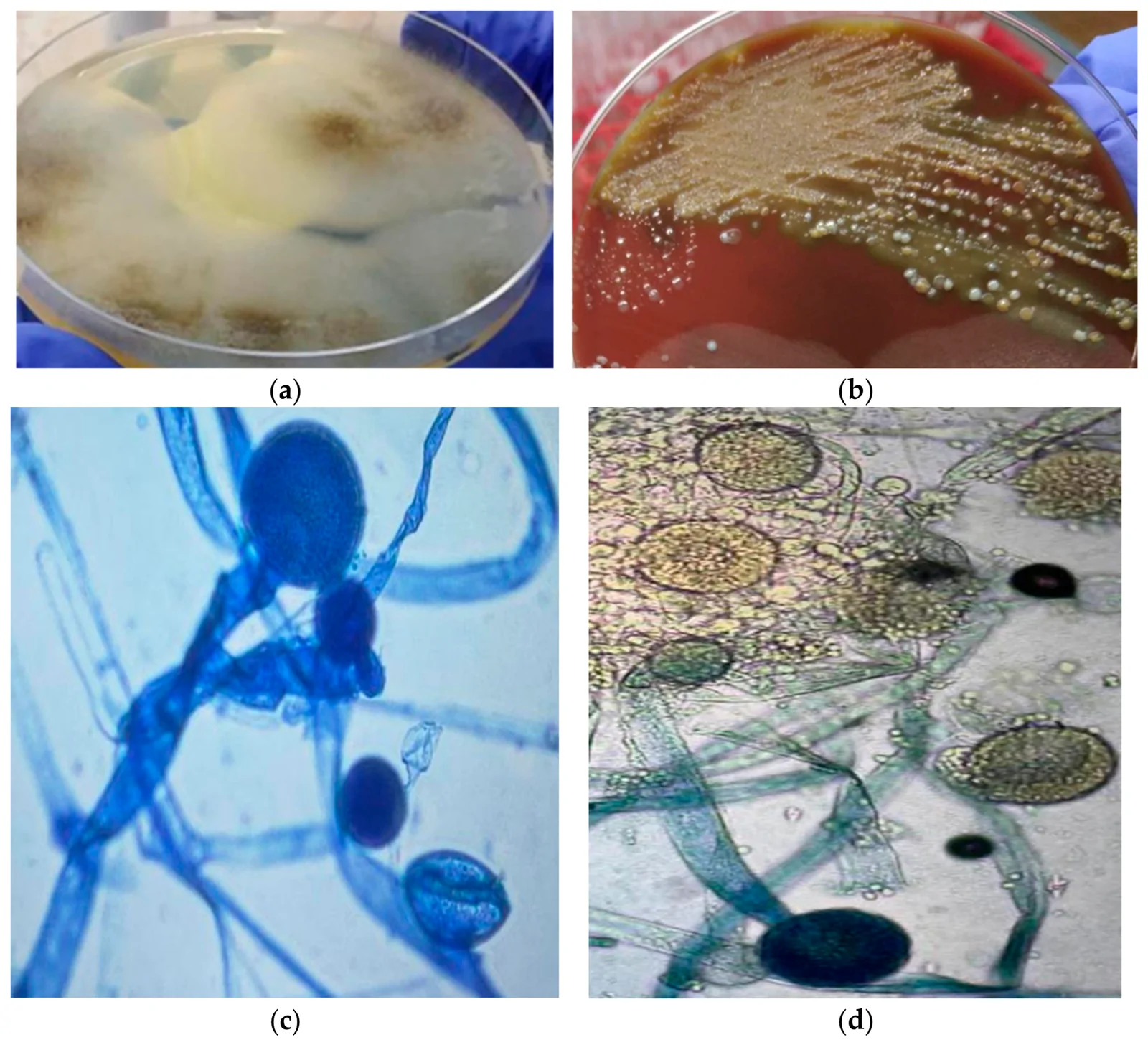
(**a**,**b**) Mold growth on an agar-based culture medium; (**c**,**d**) filamentous mycelium with a club-shaped structure at one end, observed on direct microscopic examination using a slide and coverslip preparation. The micrographs were captured under 100× magnification.

**Figure 24:**
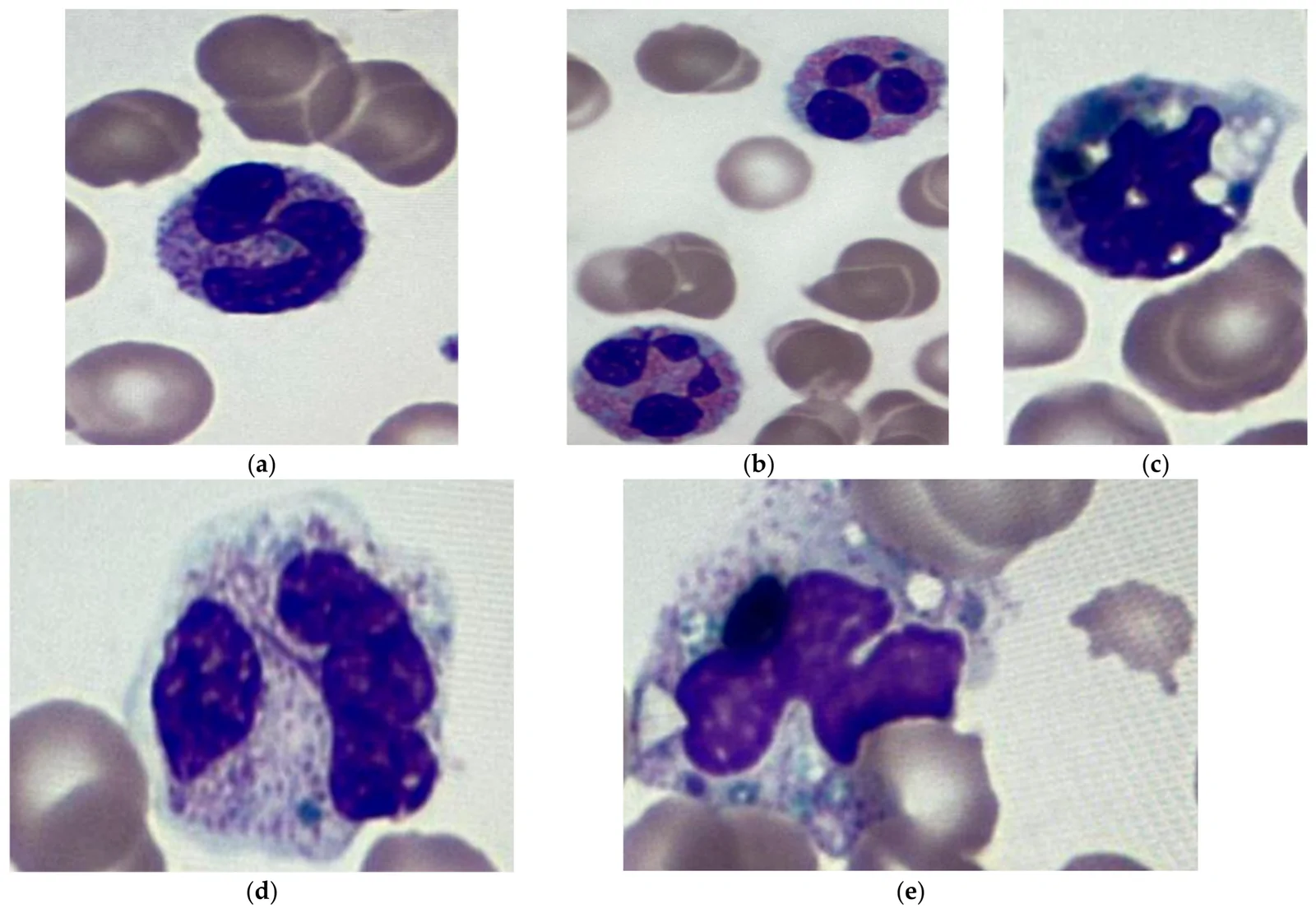
(**a**–**e**) Hematoxylin–eosin (H&E ×1000 magnification) stain of a peripheral blood smear showing large, hypergranular granulocytes with intracellular vacuoles, toxic green inclusions, and Döhle bodies, as well as schistocytes, consistent with reactive changes associated with severe invasive fungal infection in case no. 5.

**Figure 25:**
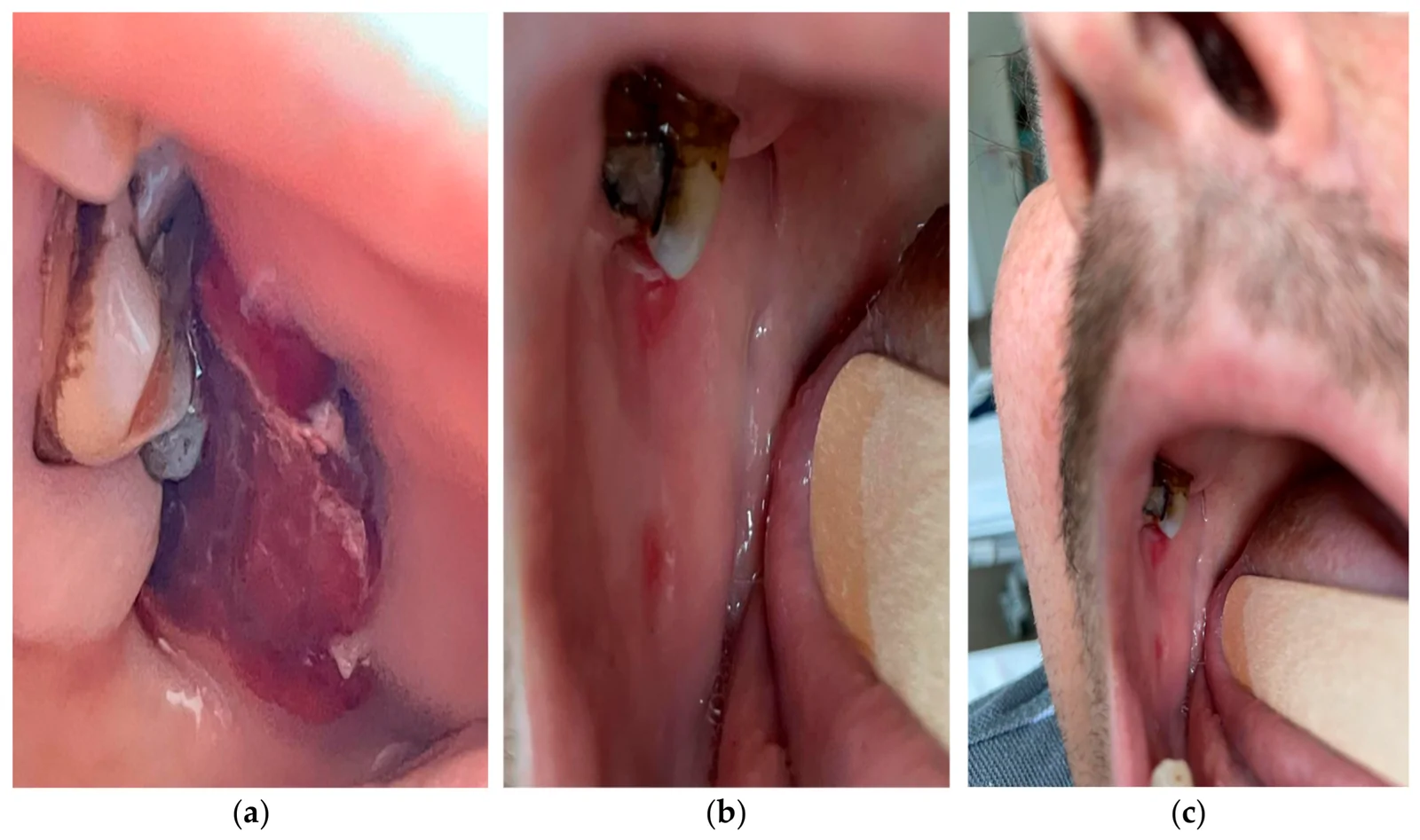
(**a**–**c**) Oral lesions involving the gingiva and buccal mucosa, associated with poor dentition, in case no. 6.

**Figure 26:**
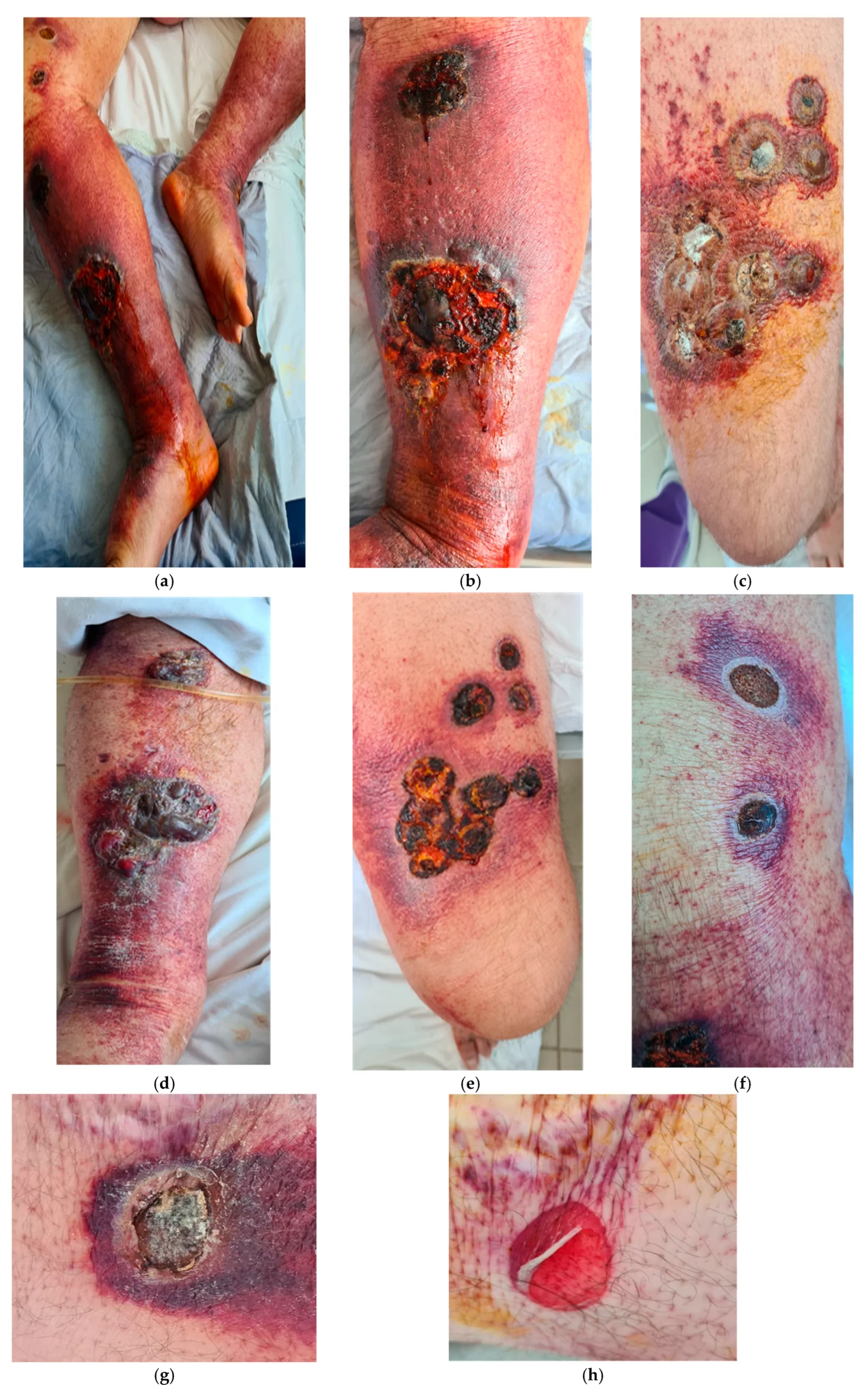
(**a**–**h**) Cutaneous mucormycosis lesions at different stages of evolution in case no. 6.

- **Five cases of rhinosino-orbito-cerebral mucormycosis:**
  ⚬ A single case of ETO-positive acute myeloid leukemia (AML), in remission during consolidation therapy with severe aplasia, associated with insulin-dependent diabetes mellitus and a rural living environment, treated with voriconazole—diagnosed on 19 June 2013, with death due to mucormycosis on 27 June 2013 (case no. 2; Figure 10, Figure 11, Figure 12, Figure 13 and Figure 14).
  ⚬ A single case of abdominal diffuse large B-cell lymphoma (DLBCL), with colostomy, during consolidation therapy in the aplastic phase, associated with insulin-dependent diabetes mellitus, treated with voriconazole—mucormycosis detected on 22 June 2021, with death due to disease progression on 7 June 2022 (case no. 3; Figure 15 and Figure 16).
  ⚬ A single case of cholangiocarcinoma undergoing chemoimmunotherapy and dendritic cell vaccination, with recent dental procedures—mucormycosis diagnosed on 20 July 2023; death due to disease progression on 30 June 2025 (case no. 1).
  ⚬ A single case of B-cell acute lymphoblastic leukemia (ALL) during chemotherapy, with severe aplasia, treated with voriconazole—diagnosed on 21 March 2024, with death due to mucormycosis on 27 March 2024 (case no. 4).
  ⚬ A single case of high-risk TP53-positive, FLT3-positive acute monoblastic leukemia, with cutaneous onset of mucormycosis on 7 October 2024, progressing to the rhinosino-orbito-cerebral form on 11 November 2024—death due to mucormycosis on 20 December 2024 (case no. 7; Figure 17, Figure 18, Figure 19, Figure 20 and Figure 21)
- **One case of pulmonary mucormycosis:**
  ⚬ A patient with T-cell non-Hodgkin lymphoma (NHL) in remission, following peripheral stem cell (PSC) collection, hospitalized for persistent respiratory syncytial virus (RSV) infection and pulmonary bacterial superinfection, treated with prophylactic voriconazole—mucormycosis diagnosed on 11 March 2024, with death due to mucormycosis on 18 March 2024 (case no. 5; Figure 22, Figure 23 and Figure 24).
- **Two cases of cutaneous mucormycosis:**
  ⚬ The disease was observed in two patients: one with acute myelomonocytic leukemia following a myelodysplastic syndrome, associated with oropharyngeal and gingival lesions superinfected with mucormycosis on 24 June 2021, medically controlled, with death due to acute myocardial infarction on 1 August 2021 (case no. 6; Figure 25 and Figure 26), and one with high-risk acute monoblastic leukemia, del(17p)-positive and FLT3-positive, with cutaneous onset of mucormycosis progressing to the rhinosino-orbito-cerebral form. The patient had insulin-dependent diabetes mellitus and a rural living environment (case no. 7) both cases were in aplasia and receiving voriconazole prophylaxis.
- **One case of urinary mucormycosis:**
  ⚬ A patient with multiple myeloma who had undergone transplantation in 2023. (case no. 8).

The analysis of the collected data yielded the following results (Table 1, Table 2 and Table 3):

## 4. Discussion

Mucormycosis, caused by fungi of the order Mucorales, is a rapidly progressive and frequently fatal invasive fungal infection first described in humans by Arnold Paltauf in 1885 [14]. Although uncommon, its burden has become increasingly apparent. Population-based surveillance in the San Francisco Bay Area during 1992–1993 estimated an annual incidence of approximately 1.7 cases per million inhabitants [23,24], while U.S. inpatient data identified 555 mucormycosis-related hospitalizations between 2005 and 2014 and an estimated 1140 hospitalizations in 2019 [24,25]. The disease occurs predominantly in immunocompromised individuals, including hematopoietic stem cell and solid organ transplant recipients, in whom it accounts for approximately 8% and 2% of invasive fungal infections, respectively [24,26]. In oncologic populations, mucormycosis is reported mainly in patients with hematologic malignancies, whereas association with solid tumors is much less frequent [9,12,27,28,29,30]. Infection usually follows inhalation of environmental spores, which may germinate into invasive hyphae and extend from the paranasal sinuses to the orbit or brain by direct spread and angioinvasion. Vascular invasion causes thrombosis, tissue ischemia, necrosis, and infarction. Clinical forms include rhino-orbito-cerebral, pulmonary, cutaneous, gastrointestinal, disseminated, and, rarely, urinary disease. Mortality remains high and varies with site and host factors, reaching 50–85% in rhino-cerebral disease, up to 90% in pulmonary disease, and extremely high levels in gastrointestinal or disseminated infection. Major predisposing factors include neutropenia, diabetes mellitus and metabolic acidosis, transplantation, advanced malignancy, corticosteroid exposure, increased iron availability, and cutaneous trauma. Neutropenia is particularly important in hematologic patients because of the central role of neutrophils in antifungal defense. Voriconazole and echinocandins do not provide adequate activity against Mucorales, and prior voriconazole exposure may contribute to the emergence of mucormycosis in highly immunosuppressed hosts. Its incidence has risen in parallel with the growing number of immunocompromised patients, reaching approximately 1% in some acute leukemia series. Cutaneous disease has been linked to trauma, including injuries associated with natural disasters, such as tsunamis and hurricanes, and more recently with SARS-CoV-2 infection during the COVID-19 pandemic and unusual presentations such as isolated urinary tract involvement have also been described. These observations are relevant to our cohort because they emphasize that mucormycosis cannot be approached as a single-site infection and that host context strongly influences clinical expression. PCR detection of circulating Mucorales DNA may support earlier diagnosis, but it is not routinely available; therefore, diagnosis often begins with clinical suspicion and imaging and is later confirmed microbiologically or histopathologically. Management rests on four principles: early recognition, prompt active antifungal treatment, correction of predisposing factors, and surgical debridement when feasible. In the index case, previous institutional experience and rapid multidisciplinary assessment facilitated recognition, and isavuconazole was started immediately because amphotericin B, considered first-line therapy, was unavailable. Classical risk factors such as neutropenia, hyperglycemia, prior voriconazole exposure, and iron overload were not evident in this patient; instead, combined chemoimmunotherapy, radiotherapy, immunomodulatory treatments, and impaired hepatic function were considered likely contributors to susceptibility. Corticosteroids were discontinued after three days and oncologic treatment was temporarily suspended. Surgical debridement is an important component of therapy, although severe cytopenias or anatomical extension may limit its feasibility; orbital exenteration has been required in advanced rhino-orbital disease. Hyperbaric oxygen has been proposed as an adjunct, but evidence remains limited, and there is no robust evidence for a direct protective or therapeutic effect of ozone therapy against mucormycosis.

Across our eight cases, mucormycosis occurred in one patient with intrahepatic cholangiocarcinoma and seven patients with hematologic neoplasms, including acute myeloid leukemia with t(8;21), acute monoblastic leukemia with del(17p), transformed follicular lymphoma to DLBCL, pre-B acute lymphoblastic leukemia, MEITL, MDS-EB2 evolving to high-risk FLT3-positive acute monoblastic leukemia, and multiple myeloma after autologous transplantation. This biological diversity was accompanied by markedly heterogeneous presentations and outcomes, reflecting the interaction between underlying disease, treatment intensity, and host immune status. Five episodes had rhino-sino-orbito-cerebral involvement, one was pulmonary, two included cutaneous disease, and one involved the urinary tract; these categories were not mutually exclusive because one cutaneous case progressed to rhino-sino-orbito-cerebral disease. Courses ranged from localized infection to ophthalmoplegia, optic nerve involvement, cerebral ischemic or hemorrhagic complications, disseminated intravascular coagulation, multiorgan failure, and death. The pulmonary case was particularly difficult to recognize because prolonged RSV infection, bacterial superinfection, organ dysfunction, and broad-spectrum antimicrobial exposure obscured the fungal process. The urinary case, with nonspecific manifestations, further illustrates that mucormycosis may remain clinically subtle even in profoundly immunocompromised patients. Within the rhino-orbital group, evolution also varied markedly: Case 2 progressed from unilateral eyelid edema to complete ophthalmoplegia, optic nerve involvement, multifocal cerebral ischemic lesions, and death, whereas Case 3 remained comparatively localized, with minimal retro-orbital involvement and rapid resolution under combined antifungal treatment. These contrasts illustrate why the clinical course cannot be inferred from anatomical classification alone. A striking feature was the temporal clustering of diagnoses. The first documented rhino-sino-orbito-cerebral case occurred in 2013, whereas seven of the eight cases were identified after 2020. In later cases, particularly Cases 1 and 3, clinical suspicion was raised within 12–24 h and often before microbiological confirmation; in Case 1, treatment was initiated approximately 13 h after symptom onset. By contrast, the early AML case progressed rapidly and ended in death within eight days despite radiologic suspicion within two days and prompt treatment. This contrast suggests that accumulated institutional experience lowered the threshold for considering mucormycosis and accelerated diagnostic pathways, although disease severity and host factors clearly remained important. In our centre, this experience changed practice: unilateral orbital symptoms, cranial nerve deficits, rapidly progressive facial swelling, and new sinus, pulmonary, or cutaneous lesions during voriconazole exposure now prompt early consideration of mucormycosis. The multidisciplinary pathway used in the cholangiocarcinoma case—integrating hematology, infectious diseases, radiology, ophthalmology, neurology, dentistry, oral and maxillofacial surgery, and intensive care—has subsequently become the institutional model for suspected disease. Earlier cases were more often characterized by advanced disease at recognition and limited therapeutic options, whereas later cases benefited from heightened awareness and faster coordination. A central feature of this series was the combined effect of severe aplasia, metabolic disturbances, and prior antifungal exposure, with profound neutropenia, thrombocytopenia, diabetes/hyperglycemia, and previous voriconazole use frequently accompanying rapidly progressive disease. In contrast, the cholangiocarcinoma patient developed mucormycosis without neutropenia or diabetes, suggesting that prolonged chemoimmunotherapy, radiotherapy, and immunomodulatory treatment may create a distinct therapy-induced immunosuppressive profile and that risk is not confined to classical hematologic settings. Clinical implications and practice changes in our center: mucormycosis is now systematically considered in the differential diagnosis of unilateral orbital symptoms, cranial nerve deficits, or rapidly progressive facial swelling in patients with hematologic malignancies, irrespective of fever or inflammatory markers. In patients receiving voriconazole who develop new sinus, orbital, pulmonary, or cutaneous lesions, breakthrough mucormycosis is actively considered, and early escalation to isavuconazole or amphotericin B is preferred over continuation or intensification of azole therapy.

The host characteristics of our patients are consistent with established risk settings, including hematologic malignancy, transplantation, diabetes mellitus, and corticosteroid exposure [11,14,24,31]. Corticosteroids are a recognized predisposing factor [28,32,33,34], while acute leukemia, prolonged neutropenia, and hematopoietic stem cell transplantation remain major hematologic settings [12,35]. Relapsed or refractory disease, prolonged neutropenia, and corticosteroid exposure also contribute to the cumulative risk of invasive mould infections [31,35,36,37,38,39,40], particularly when several factors coexist [41,42,43]. Although evidence from invasive mould infections overall cannot be fully extrapolated to mucormycosis [24,40,42], our cases similarly demonstrate cumulative immunosuppression related to chemotherapy, corticosteroids, neutropenia, metabolic abnormalities, and other treatment-related factors [35,41,42,43]. At mucormycosis onset, most hematologic patients were in severe aplasia, with neutrophil counts of 0–700/mm^3^ and frequent profound thrombocytopenia that restricted surgery. Diabetes or hyperglycemia of approximately 180–230 mg/dL was present in several cases. The cholangiocarcinoma patient, however, developed mucormycosis without neutropenia or diabetes, suggesting that prolonged chemoimmunotherapy, radiotherapy, and immunomodulatory treatment can produce a different but clinically relevant immunosuppressive profile. This single observation cannot be generalized, but it supports maintaining diagnostic awareness beyond classical hematologic settings. In several hematologic cases, hyperglycemia coexisted with profound aplasia, potentially amplifying susceptibility and complicating recovery. Prior voriconazole exposure was another recurrent feature; together with corticosteroid exposure, iron overload, and environmental factors, this may have contributed to a clinical context favorable to Mucorales. The important point is not a single isolated risk factor but the cumulative immunologic and metabolic burden carried by these patients. From a clinical perspective, our findings support extending the level of suspicion traditionally applied to hematologic malignancies to other oncologic settings characterized by profound or prolonged immunosuppression. In particular, clinicians managing patients with solid tumors undergoing intensive multimodal treatment, including chemotherapy and immunomodulatory therapies, should maintain awareness of mucormycosis as a potential diagnosis, especially in the presence of additional risk factors such as corticosteroid exposure or neutropenia. Finally, the temporal distribution of cases in our series, with the majority diagnosed after 2020, parallels the increase in mucormycosis incidence reported globally in recent years, including in the context of COVID-19-related immune dysregulation and wide-spread corticosteroid use. While causality cannot be inferred from our data, this observation reinforces the need for heightened clinical vigilance in the current epidemiological context [11,31,33,44,45].

The anatomical distribution in our series also differed from many large hematologic cohorts, in which pulmonary disease generally predominates [12,33], including recent hematologic and intensive-care studies [12,35,36]. Our patients showed substantial extrapulmonary involvement, particularly rhino-sino-orbito-cerebral disease, together with uncommon cutaneous and urinary manifestations [12,28,33,46,47,48]. In the index case, orbital involvement initially raised concern for metastatic disease, illustrating the diagnostic overlap between infectious, inflammatory, and neoplastic orbital lesions [28,49,50]. Urinary involvement, which is rarely reported, likewise demonstrates that mucormycosis may occur outside the thoracic and craniofacial sites most often investigated [28,46,48]. Diagnostic evaluation in high-risk patients should therefore follow the clinical presentation rather than being restricted to pulmonary imaging [47]; absence of pulmonary abnormalities does not exclude invasive fungal infection [28,46], and unusual manifestations warrant a site-oriented approach [33]. This is especially relevant in oncologic patients, in whom orbital, cutaneous, or urinary abnormalities may initially be attributed to tumor progression, bacterial infection, treatment toxicity, or other complications. A flexible anatomical approach is therefore necessary to avoid systematically under-recognizing extrapulmonary disease.

Diagnostic delay is consistently associated with adverse outcome in mucormycosis [31,36,51,52]. Published series have reported delays of up to 26 days, including diagnoses made only post-mortem [51], while ICU cohorts describe a median interval of approximately seven days [36]; delays exceeding 16 days have been independently associated with mortality [52]. In hematologic malignancy-associated zygomycosis, amphotericin B-based therapy initiated ≥6 days after symptom onset was associated with a twofold increase in mortality [53]. Conventional culture and histopathology can further delay confirmation because of limited sensitivity and turnaround times, with culture requiring up to 3–7 days and histopathology approximately 48–72 h [31,35,51,54,55,56]. These limitations support the current emphasis on early clinical suspicion [11,12,31]. In our index case, antifungal therapy was initiated approximately 13 h after symptom onset based on clinical and imaging findings, before microbiological confirmation, consistent with recommendations for prompt treatment when mucormycosis is suspected [31,57]. CT and MRI were central to early site-specific assessment [31,57]. Similar early treatment occurred in selected later cases. Although our small retrospective series cannot establish that a specific 24-h threshold improves survival, it demonstrates the feasibility of a suspicion-driven strategy and is consistent with larger studies linking diagnostic and therapeutic delay to poor outcome [35,52,53]. Thus, in high-risk patients with rapidly progressive or atypical syndromes, treatment decisions may need to rely on host factors, clinical presentation, and imaging rather than waiting for definitive microbiological proof [31,36,51,57]. In selected patients in our series, antifungal therapy was instituted within the first 12–24 h of suspicion, before microbiological confirmation. The favorable evolution observed in some of these cases should not be interpreted as causal evidence from this small series, but it illustrates how a rapid diagnostic pathway can be operationalized in routine practice and is consistent with the broader literature on the prognostic importance of treatment delay.

Breakthrough mucormycosis during antifungal exposure is increasingly recognized in patients with hematologic malignancies [13,58]. It may develop during therapy with agents lacking activity against Mucorales, particularly voriconazole, and less frequently during posaconazole, isavuconazole, or amphotericin B treatment [58]. Prognosis is especially poor when profound immunosuppression and unresolved neutropenia persist [12,58,59]. Voriconazole is particularly relevant because it lacks activity against Mucorales [58,60,61]. Several patients in our series developed mucormycosis despite prior antifungal exposure, and voriconazole was discontinued once mucormycosis was suspected. Persistent, progressive, or atypical lesions during triazole prophylaxis or treatment should therefore prompt diagnostic reassessment rather than continuation of the same regimen without reconsidering the underlying aetiology [52,53]. This approach is consistent with recommendations for infections arising during voriconazole exposure [31,61] and is especially important in hematologic patients, in whom deterioration may initially be attributed to malignancy or concomitant infections [13,62,63]. Breakthrough disease reflects the interaction between host immunosuppression, pathogen biology, and the limitations of antifungal coverage rather than prophylaxis failure alone. Accordingly, ongoing antifungal prophylaxis should not create a false sense of security. In patients with unexplained clinical deterioration or new radiological lesions, prior antifungal exposure should shape the differential diagnosis and therapeutic reassessment but should never reduce vigilance for mucormycosis.

Treatment in our series followed the principle that effective management requires coordinated antifungal therapy, surgical assessment, and correction of modifiable host factors [31,61,64,65]. Liposomal amphotericin B remains a principal first-line agent [31,61], while treatment must be adjusted according to clinical, radiological, and microbiological evolution [61,65]. Earlier cases often received liposomal amphotericin B with posaconazole, whereas isavuconazole was used more extensively in later cases, including as monotherapy when amphotericin B was unavailable. Prolonged therapy, reaching six months in selected patients, was associated with complete clinical and radiological resolution in Cases 1 and 3. Surgical debridement was pursued when feasible [31,36,66], but aplasia, severe thrombocytopenia, coagulation abnormalities, anatomical extension, and clinical instability frequently limited intervention. Correction of underlying factors—including neutrophil recovery, reduction or discontinuation of corticosteroids, glycemic control, removal of infected devices, and management of comorbidities—was integral to care [12,31,59,61]. Rapid coordination among hematology, infectious diseases, radiology, intensive care, and site-specific surgical specialties allowed treatment and surgical decisions to be adapted as disease evolved. This time-sensitive multidisciplinary organization may reduce avoidable delays and operationalizes the core principles emphasized in international recommendations [31,61,65,66]. In practice, management had to remain individualized: critically ill patients sometimes deteriorated too rapidly to sustain planned antifungal combinations or undergo surgery, whereas clinically stable patients could receive prolonged treatment and serial imaging. These differences reinforce that successful care depends not only on the antifungal agent selected but also on the ability to coordinate supportive care, local source control, and recovery of host defenses.

Reported mortality in hematology and HSCT populations ranges from approximately 40% to 80% [35,67], and may approach 70% in critically ill cohorts [36]. Non-recovered neutropenia, corticosteroid exposure, disseminated disease, and delayed appropriate therapy are repeatedly associated with poor outcome [35,53,67], while breakthrough mucormycosis also carries an unfavorable short-term prognosis [58]. Our observations were consistent with these patterns: persistent aplasia, thrombocytopenia, multiple infections, septic shock, disseminated intravascular coagulation, extensive disease, and multiorgan dysfunction characterized unfavorable courses, whereas clinical improvement more often accompanied hematologic recovery, control of modifiable factors, and timely active antifungal treatment [35,53,67]. The comparison between Cases 1 and 2 illustrates that diagnostic timing cannot be considered in isolation. Case 2 deteriorated rapidly despite relatively prompt recognition, in the setting of severe aplasia and metabolic risk factors, whereas Case 1 had no aplasia and achieved complete resolution after suspicion at approximately 13 h and immediate targeted therapy. Thus, prognosis in this series appears to reflect the cumulative interaction of host status, disease extent, and timing of intervention rather than any single variable. Given the small sample, these associations are descriptive and do not establish independent prognostic effects [35,36,52,67]. Nevertheless, across the series the most clinically actionable factors were consistent: maintain early suspicion, initiate active antifungal therapy without unnecessary delay, correct reversible risk factors, and pursue local intervention whenever the hematologic and anatomical situation permits. Poor outcomes occurred particularly when several adverse factors converged, supporting the concept that prognosis is driven by cumulative host- and disease-related severity rather than by a single determinant.

Seven of eight cases were diagnosed after 2020, paralleling increased reporting of mucormycosis during the COVID-19 pandemic, particularly COVID-19-associated mucormycosis [11,32,68]. Proposed contributors include systemic corticosteroid use, immune dysregulation, and worsening or newly recognized diabetes mellitus [32,41,42,43], while intensified immunosuppressive therapies and prolonged hospitalizations may also have influenced the epidemiology of invasive fungal infections. Because our cohort was not restricted to COVID-19-associated disease, this temporal clustering cannot be attributed specifically to SARS-CoV-2. It should instead be interpreted in the context of broader epidemiological changes among highly immunocompromised populations [11,31,33,44,45], reinforcing the need for continued vigilance in the post-pandemic period.

A particularly important contribution of this series is the occurrence of rhino-sino-orbital mucormycosis in a patient with established intrahepatic cholangiocarcinoma after prolonged multimodal oncologic treatment. To our knowledge, mucormycosis complicating an established cholangiocarcinoma has not previously been reported. Conversely, hepatic mucormycosis involving the biliary tract has been described as mimicking hilar cholangiocarcinoma [8], illustrating the reverse diagnostic relationship. In contrast to these reports, our case demonstrates the reverse scenario, in which a known malignancy obscured the recognition of an invasive fungal infection and demonstrates how invasive fungal infection can be obscured by an expected oncologic differential. More broadly, our cohort adds case-level detail to the recognized heterogeneity of mucormycosis. Pulmonary and rhino-orbital/sino-facial disease predominate in larger hematologic series [9,12,47], whereas cutaneous and urinary mucormycosis remain uncommon, and rhino-orbito-cerebral disease requires rapid diagnosis, active antifungal treatment, and surgical evaluation when feasible [31,45,69,70,71,72,73], but severe hematologic abnormalities frequently restricted surgery in our patients. The short diagnostic intervals in selected cases illustrate the feasibility—not proof of efficacy—of a suspicion-driven approach [31,61], particularly given delays reported elsewhere [36,51] and the association between delayed amphotericin B-based treatment and mortality [26,53]. Clinical and imaging findings can therefore support early decisions in high-risk patients [26,74], especially in rapidly progressive rhino-orbito-cerebral disease [75]. Likewise, disease developing despite prior mould-active exposure is consistent with reports of breakthrough mucormycosis [58,76,77] and remains a challenge in hematologic malignancies [58,78,79,80,81,82], requiring reassessment when infection progresses during therapy [58,76]. Taken together, our experience provides a practical framework linking early suspicion, site-directed imaging, rapid antifungal initiation, correction of host factors, and multidisciplinary decision-making across both classical and atypical presentations. In contrast to larger datasets that primarily provide aggregate outcomes, our series documents the sequence of symptom onset, antifungal exposure, imaging evolution, surgical feasibility, and clinical response at case level. This detail helps illustrate the bedside diagnostic traps encountered in patients with complex malignancies, particularly when mucormycosis resembles oncologic progression or develops despite antifungal prophylaxis. The series therefore contributes not by establishing new causal relationships, but by showing how established principles can be applied under real-world constraints.

This study has several strengths, including detailed case-level documentation of eight episodes from symptom onset through clinical suspicion, diagnostic work-up, imaging, antifungal exposure, treatment, surgical decisions, and multidisciplinary management. Inclusion of hematologic malignancies and a solid-tumor case, together with uncommon cutaneous and urinary manifestations, broadens the represented clinical spectrum. However, the retrospective single-centre design and small sample limit generalizability and preclude formal prognostic analysis. Selection bias is possible because only clinically overt, microbiologically or histologically confirmed cases were included, and the observational design does not permit causal inference regarding early suspicion, specific antifungal regimens, multidisciplinary management, or surgery. These findings should therefore be interpreted as descriptive observations consistent with established evidence and current recommendations, and larger multicentre studies are needed to confirm and extend them. Additional integrated, detailed, and in-depth information regarding the detailed clinical, microbiological, laboratory, therapeutic, and outcome data for the eight patients with mucormycosis, as well as the detailed oncologic history, therapeutic lines, and exposure for the eight patients, can be found in Appendix A, Table A1 and Table A2. 

## 5. Conclusions

Mucormycosis remains a rapidly progressive and life-threatening infection in immunocompromised patients, particularly in those with hematologic malignancies and complex oncologic backgrounds. Our series shows that the clinical spectrum of mucormycosis in this population extends beyond classical pulmonary presentations and may include atypical anatomical sites such as rhino-sino-orbital, cutaneous, and urinary involvement, which can complicate and delay diagnosis. In addition, the occurrence of infection despite prior antifungal exposure underscores that prophylaxis does not eliminate risk in highly immunosuppressed patients.

Our experience supports a suspicion-driven approach in high-risk patients, in which early recognition based on clinical and imaging features may allow initiation of antifungal therapy before microbiological confirmation is available. In our series, this approach was combined with rapid multidisciplinary management and correction of underlying risk factors; however, the small retrospective nature of the series does not allow conclusions regarding its efficacy or impact on mortality.

Overall, our findings support maintaining a high index of suspicion for mucormycosis in high-risk patients, including those with atypical presentations or prior antifungal exposure, and are consistent with current recommendations favoring prompt antifungal treatment when mucormycosis is clinically suspected.

## Figures and Tables

**Table 1:** Baseline characteristics and major risk factors of the eight patients with mucormycosis.

Parameter	Case 1	Case 2	Case 3	Case 4	Case 5	Case 6	Case 7	Case 8
Sex	Female	Male	Male	Male	Male	Male	Male	Male
Age at mucormycosis onset, years	64	49	54	56	61	52	47	53
Underlying disease	Cholangiocarcinoma	AML with maturation, t(8;21), ETO-AML+	Follicular lymphoma transformed to CD20+ large B-cell lymphoma	Pre-B ALL	MEITL/EATL type II	MDS-EB2 evolved to FLT3+ AML	Acute monocytic leukemia (AML M5a), del(17p)	IgG kappa multiple myeloma
Disease category	Solid tumor	Hematologic	Hematologic	Hematologic	Hematologic	Hematologic	Hematologic	Hematologic
Residence	Urban	Rural	Urban	Urban	Urban	Rural	Rural	Urban
Aplasia at onset	No	Yes	Yes	Yes	No	Yes	Yes	No
Days of aplasia before mucormycosis	NA	17	14	5	NA	21	12 days after first course; 5 days after second course	NA
Interval from last therapy to onset	24 days after nivolumab; 45 days after IO-VAC booster	17 days after HDAC + anthracycline	14 days after DHAP	5 days after HyPER-CVAD block B	64 days after DHAP and stem cell collection	21 days after “3 + 7 + midostaurin”	12 days after “3 + 7”; cutaneous mucormycosis5 days after HDAC + etoposide + idarubicin. Rhinosino-orbito-cerebral mucormycosis.	3 years after KRd
Blood glucose, mg/dL	87	183	125	183	230	225	122	120
Neutrophils, /mm^3^	5080	460	700	0	7480	200	30 at first episode; 10 at second orbital episode	Within normal range
Major comorbidities/risk factors	Hepatocytolysis; cholestasis; hypothyroidism; chemotherapy; radiotherapy; immunotherapy; dental procedures; disease in partial remission.	Decompensated diabetes mellitus; corticosteroid use; prophylactic voriconazole; hypersideremia; neutropenia; prolonged hospitalization.	Insulin-dependent diabetes; corticosteroids; prophylactic voriconazole, neutropenia Colostomy; disease in partial remission.	Corticosteroids; voriconazole exposure Morphologic complete remission with MRD positivity.	Prolonged RSV infection; bacterial superinfection; corticosteroids; voriconazole exposure; steroid-induced hyperglycemia	Insulin-dependent diabetes; corticosteroids; voriconazole; neutropenia prolonged hospitalization; active disease.	Corticosteroid therapy; voriconazole exposure; prolonged hospitalization; farm exposure.	Humoral immune deficiency; rural/spore-rich exposure; therapy.

Abbreviations: AML, acute myeloid leukemia; ALL, acute lymphoblastic leukemia; DHAP, dexamethasone/cytarabine/cisplatin; HDAC, high-dose cytarabine; KRd, carfilzomib/lenalidomide/dexamethasone; MDS, myelodysplastic syndrome; MEITL, monomorphic epitheliotropic intestinal T-cell lymphoma; NA, not applicable.

**Table 2:** Underlying oncologic treatment and relevant exposure prior to mucormycosis.

Parameter	Case 1	Case 2	Case 3	Case 4	Case 5	Case 6	Case 7	Case 8
Oncologic treatment summary before mucormycosis	Hepatectomy; GEM/Oxaliplatin-based chemotherapy; nivolumab; SIRT; IO-VAC; multiple adjunct experimental therapies	“3 + 7” induction; 4 HDAC + anthracycline; relapse/re-“3 + 7” induction; 3 HDAC + anthracycline induction; stem cell donor work-up	CHOP-R debulking; DHAP-R	HyPER-CVAD induction, then block B with HD-MTX + HD-Ara-C	Surgery; 6 CHOEP; 2 DHAP; stem cell collection	Azacitidine for MDS; hydroxyurea; “3 + 7 + midostaurin” for AML	“3 + 7” induction; salvage HDAC + etoposide + idarubicin	Bortezomib/dexamethasone/liposomal doxorubicin; autologous SCT; CyBorDex; KRd; radiotherapy for costal plasmacytoma.
Last relevant antineoplastic exposure before mucormycosis	Nivolumab	HDAC + anthracycline	DHAP	HyPER-CVAD block B	DHAP/stem cell collection	“3 + 7 + midostaurin”	Post-induction aplasia after “3 + 7”; later after salvage chemotherapy	No recent cytotoxic therapy; prior KRd long before event
Immunotherapy/targeted therapy	Nivolumab	No	Rituximab-containing regimens	No	No	Midostaurin	No	
Radiotherapy/locoregional therapy	SIRT (Y-90)	No	No	No	No	No	No	Radiotherapy to rib plasmacytoma
Experimental/non-standard adjuncts	ICD, mEHT, NDV, WBH, IO-VAC, DC vaccine, ozone therapy	No	Hyperbaric therapy later during treatment of mucormycosis	No	No	Topical amphotericin, local wound/oral care later during treatment	Extensive surgical drainage/debridement later during treatment	No
Recent procedure/exposure of interest	Dental procedures	None specified	Contact with another patient with cutaneous mucormycosis	Prior infectious complications during aplasia	Prolonged hospitalization; stem cell collection; RSV-related complications	None specified	Farm residence; repeated hospitalizations; central venous catheter	Travel to spore-rich environment
Antifungal exposure before mucormycosis	None stated	Voriconazole prophylaxis	Voriconazole prophylaxis	Voriconazole prophylaxis/treatment	Voriconazole exposure	Voriconazole exposure	Voriconazole exposure	No prior antifungal exposure
Main background conditions favoring infection	Cancer-related immune dysregulation	Post-chemotherapy aplasia + uncontrolled diabetes	Aplasia + insulin-dependent diabetes + steroids	Profound aplasia + steroids	Polychemotherapy + prolonged viral/bacterial infection + hyperglycemia	Aplasia + uncontrolled diabetes + steroids	Repeated profound aplasia + mixed bacterial/fungal colonization	Humoral immune dysfunction

Abbreviations: DRd, daratumumab/lenalidomide/dexamethasone; HD-MTX, high-dose methotrexate; HD-Ara-C, high-dose cytarabine; ICD, immunogenic cell death; IO-VAC, autologous antitumor vaccine; mEHT, modulated electrohyperthermia; NDV, Newcastle disease virus; SIRT, selective internal radiotherapy; WBH, whole-body hyperthermia.

**Table 3:** **Clinical presentation, diagnosis, treatment, and outcomes of mucormycosis.**

Parameter	Case 1	Case 2	Case 3	Case 4	Case 5	Case 6	Case 7	Case 8
Form of mucormycosis	Rhino-sino-orbital	Rhino-sino-orbito-cerebral	Rhino-sino-orbital	Rhino-sino-orbito-cerebral	Pulmonary	Cutaneous and oral	Cutaneous, then rhino-sino-orbito-palato-buccal with cerebral spread	Urinary
Main presenting features	Eyelid edema, chemosis, ocular pain, diplopia, ptosis, exophthalmos Figure 5a–d	Eyelid edema, ptosis, exophthalmos, visual loss, ophthalmoplegia Figure 10, Figure 11 and Figure 12	Conjunctival congestion, eyelid edema, ocular pain, exophthalmos, ptosis Figure 15	Facial asymmetry, exophthalmos, ptosis, facial palsy; fulminant progression	Worsening respiratory failure; fever; disseminated intravascular coagulation (DIC); multiple organ failure (MOF); resuscitation discontinued.	Oropharyngeal, gingival, palatal, and cutaneous lesions Figure 25 and Figure 26	Necrotic calf lesion; later exophthalmos, nasal/orbital progression, palatal necrosis, cerebral hemorrhage Figure 17	Urinary and upper respiratory symptoms
Imaging summary	Retroorbital nodular lesion; minimal sinonasal mucosal thickening Figure 6a–d, Figure 7 and Figure 9	Orbital fat infiltration, sinusitis, optic nerve involvement; possible cerebral lesions Figure 13	Minimal retroorbital infiltration; ethmoid-sphenoid rhinosinusitis Figure 16	MRI: sphenoid/mastoid/frontal mucosal thickening with eyelid edema	CT: pulmonary consolidation with air bronchogram and pleural effusion Figure 22	Brain CT normal	Sinus/orbital progression on CT/MRI; later cerebral lesion/hemorrhage Figure 18, Figure 19, Figure 20 and Figure 21	No relevant imaging abnormalities
Time onset → suspicion	13 h	24 h	12 h	24 h	Not clinically suspected	Not suspected	Not initially suspected cutaneous form; 6 h during the orbital episode at second admission for chemotherapy.	No suspicion raised
Time onset → fungal isolation	48 h	36 h	24 h	96 h	Approx. 48 h from respiratory deterioration	3 days	24 h in first hospitalization; 24 h in second	24 h
Microbiological diagnosis summary	Conjunctival and nasal secretions positive for mold/Mucorales-compatible growth Figure 8A–C	Conjunctival and nasal secretions positive Figure 14	Nasal and lacrimal sac cultures positive for *Mucor* spp.	*Mucor* spp. from nasal cavity and conjunctival sac	*Mucor* spp. from sputum and pharyngeal exudate Figure 23 and Figure 24	*Mucor* spp. from cutaneous and gingivobuccal lesions	Positive cultures from cutaneous, conjunctival, oral, nasal, and sinus samples	*Mucor* spp. isolated from urine
Associated infections	*Klebsiella pneumoniae* (sputum)	Enterococcus faecium	Candida albicans; Pseudomonas aeruginosa	Leclercia adecarboxylata; Serratia liquefaciens; Pseudomonas fluorescens	Candida lipolytica; Enterococcus faecium	Pseudomonas fluorescens, Hafnia alvei, *Acinetobacter* spp.; Candida glabrata	Multiple bacterial/fungal coinfections incl. Klebsiella, Acinetobacter, Enterococcus, Pseudomonas, Aspergillus, Fusarium	Haemophilus haemolyticus; Raoultella planticola; Candida in stool
Main antifungal treatment	Isavuconazole	Amphotericin B lipid complex + posaconazole	Isavuconazole + amphotericin B; later maintenance isavuconazole	Isavuconazole	Isavuconazole + amphotericin B	Posaconazole, then isavuconazole + amphotericin B; topical amphotericin	Sequential/combined posaconazole, isavuconazole, amphotericin B	Posaconazole
Treatment duration	6 months	6 days	110 days total	6 days	Isavuconazole 7 days; amphotericin B 6 days	Hospitalized for 24 days, followed by isavuconazole treatment at home.	~30 days first episode; 1 November–20 December 2024 during second episode	10 days
Clinical/radiological outcome	Rapid recovery; near-complete radiologic resolution by 2–3 months	Progressive deterioration and death	Clinical and imaging remission	Fulminant progression and death	Unfavorable; death	Clinical and imaging remission	Initial cutaneous improvement, then rhino-orbital-palatal-cerebral progression	Remission; negative urine culture
Death due to mucormycosis	No	Yes	No	Yes	Yes	No	Yes	No
Death due to underlying disease	Yes	No RC	Yes	No	No RC	No	No RC	No
Interval mucormycosis → death	24 months	8 days	~1 year after mucormycosis, due to lymphoma progression	4 days	9 days	45 days, unrelated MI	From second invasive episode: 13 November–20 December 2024	NA
Final mucormycosis outcome	Resolved	Not resolved	Resolved	Not resolved	Not resolved	Resolved	Not resolved	Resolved
Survival status at last follow-up	Deceased	Deceased	Deceased	Deceased	Deceased	Deceased	Deceased	Alive

Abbreviations: DIC, disseminated intravascular coagulation; NA, not applicable.

## Data Availability

The original contributions presented in the case report are included in the article, further inquiries can be directed to the corresponding authors.

## Abbreviations

The following abbreviations are used in this manuscript:

SARS-CoV-2	Severe Acute Respiratory Syndrome Coronavirus 2
RHL	Right hepatic lobe
CT	Computed tomography
MRI	Magnetic resonance imaging
TNM	Tumor, Node, Metastasis
LHL	Left hepatic lobe
ICD	Immunogenic cell death
mEHT	Modulated electrohyperthermia
NDV	Newcastle disease virus
PET–CT	Positron emission tomography–computed tomography
SIRT	Selective intrahepatic radiotherapy
WBH	Whole body hyperthermia
FDG	Fluorodeoxyglucose
CT TAP	Computed tomography of the thorax, abdomen, and pelvis
WBC	White Blood Cells
Hb	Hemoglobin
CRP	C-Reactive Protein
TSH	Thyroid Stimulating Hormone
CA125	Cancer Antigen 125
ALP	Alkaline phosphatase
GGT	Gamma Glutamyl Transferase
AST	Aspartate Aminotransferase
ALT	Alanine Aminotransferase
SDA	Sabouraud dextrose agar
SBA	Sheep blood agar
CLED	Cystine–lactose–electrolyte deficient
AML	Acute myeloid leukemia
DLBCL	Diffuse large B cell lymphoma
ALL	Acute lymphoblastic leukemia
NHL	non-Hodgkin lymphoma
PSC	Peripheral stem cell
RSV	Respiratory syncytial virus
MEITL	Monomorphic epitheliotropic intestinal T-cell lymphoma
MDS	Myelodysplastic syndrome
DIC	Disseminated intravascular coagulation
H&E	Hematoxylin–eosin
COVID-19	Coronavirus Disease 2019
ENT	Ear, Nose, and Throat
ICU	Intensive Care Unit
HSCT	Hematopoietic Stem Cell Transplantation
CAM	COVID-19-associated mucormycosis

## Appendix A

**Table A1:** Detailed clinical, microbiological, laboratory, therapeutic, and outcome data for the eight patients with mucormycosis.

Parameter	Case 1	Case 2	Case 3	Case 4	Case 5	Case 6	Case 7	Case 8
Date of mucormycosis onset/suspicion	24 June 2023	19 June 2013	22 June 2021	25 March 2024	11 March 2024	24 June 2021	7 October 2024 (cutaneous), 13 November 2024 (systemic)	Not suspected
Clinical presentation (detailed)	Asthenia; hemicrania; eyelid edema; chemosis; ocular pain; diplopia; ptosis; photophobia; facial asymmetry; exophthalmos	Conjunctival congestion → ptosis → exophthalmos → blindness; ophthalmoplegia	Eyelid edema; conjunctival congestion; ocular pain; exophthalmos; ptosis	Facial asymmetry; exophthalmos; cranial nerve palsies; fulminant course with DIC and MSOF	Respiratory failure; fever; DIC; rapid deterioration	Oral, gingival, palatal, cutaneous lesions	Cutaneous lesion → orbital → palatal necrosis → cerebral hemorrhage	Mild urinary + respiratory symptoms
Imaging (detailed)	CT/MRI: retroorbital lesion, optic nerve contact, sinus mucosal thickening	MRI: Orbital infiltration, optic nerve involvement, ischemic/cerebritis lesions	MRI: minimal retroorbital inflammation; sinusitis	MRI: Sphenoid/mastoid/frontal mucosal thickening	CT: Pulmonary consolidation, pleural effusion	CT normal	Multiple CT/MRI: Progressive sinus–orbital–cerebral extension, hemorrhage	No relevant findings
Initial suspicion	13 h	24 h	12 h	24 h	Not suspected	Not suspected	Delayed; confirmed later	Not suspected
Time to fungal isolation	48 h	36 h	24 h	96 h	~48 h	3 days	24 h (both episodes)	24 h
Microbiological diagnosis (detailed)	Conjunctival + nasal cultures; mold; Sabouraud	Same	*Mucor* spp. from nasal/lacrimal	*Mucor* spp. nasal + conjunctival	*Mucor* spp. sputum + pharynx; detailed smear changes	*Mucor* spp. cutaneous + oral	Multiple sites: cutaneous, nasal, sinus, ocular	*Mucor* spp. urine
Associated infections	*Klebsiella pneumoniae*	Enterococcus faecium	Candida, Pseudomonas	Serratia, Leclercia, Pseudomonas	Candida lipolytica, Enterococcus	Pseudomonas, Acinetobacter, Candida	Multiple MDR bacteria + fungi	Haemophilus, Raoultella, Candida
Inflammatory markers	CRP 7.8	CRP 186	CRP 0.64	CRP 126; PCT ↑	CRP up to 232; PCT ↑; IL-6 ↑	CRP 11	CRP up to 211	CRP 64
CBC (detailed)	WBC 6400; neutrophils 5080; Hb 8; PLT 138k	Severe neutropenia; PLT 14k	Neutropenia; PLT 26k	WBC 10; neutrophils 0; PLT 13k	WBC 11k; neutrophilia	Neutrophils 200; PLT 23k	Profound pancytopenia → later recovery	Normal
Glycemia	87	183	125	183	230	225	122	120
Other risk factors	Hepatic dysfunction; hypothyroidism	Diabetes; steroids; voriconazole; iron overload	Diabetes; steroids; voriconazole	Steroids; voriconazole	RSV; steroids; polyinfection	Diabetes; steroids; voriconazole	Farm exposure; prolonged hospitalization	Travel exposure; immune deficit
Treatment (detailed)	Isavuconazole long-term	Amphotericin B + posaconazole	Isavuconazole + amphotericin + hyperbaric	Isavuconazole	Isavuconazole + amphotericin	Posaconazole → isavuconazole + amphotericin + topical	Amphotericin + posaconazole + isavuconazole + surgery	Posaconazole
Treatment duration	6 months	6 days	110 days	6 days	~1 week	24 days + continuation	~30+ days	10 days
Outcome (detailed)	Full recovery	Rapid death	Remission	Rapid death	Death	Remission	Progression and death	Remission
Death due to mucormycosis	No	Yes	No	Yes	Yes	No	Yes	No
Death due to underlying disease	Yes	No	Yes	No	No	No	No	No
Interval mucormycosis → death	24 months	8 days	~1 year	4 days	9 days	45 days	~1 month	NA
Final outcome	Resolved	Not resolved	Resolved	Not resolved	Not resolved	Resolved	Not resolved	Resolved
Survival	Deceased	Deceased	Deceased	Deceased	Deceased	Deceased	Deceased	Alive

**Table A2:** Detailed oncologic history, therapeutic lines, and exposure for the eight patients.

Parameter	Case 1	Case 2	Case 3	Case 4	Case 5	Case 6	Case 7	Case 8
H-O disease	Cholangiocarcinoma	AML t(8;21)	Transformed lymphoma	Pre-B ALL	MEITL	MDS → AML	AML M5a	Multiple myeloma
Date of diagnosis	2009	2010	2021	2024	2023	2019/2021	2024	2017
Therapeutic lines (FULL, preserved)	L1 hepatectomy; L2 GEM + Oxaliplatin + ICD + mEHT + NDV (PR); L3 nivolumab + GEM + ICD + mEHT + NDV (CR→PD); L4 SIRT; L5 IO-VAC; L6 nivolumab + WBH + NDV + others	L1 “3 + 7” → CR; consolidation HDAC; L2 relapse → re-induction → CR	CHOP-R → PR; DHAP-R	HyPER-CVAD → MRD + ; Block B HD-MTX + Ara-C	Surgery; CHOEP; DHAP; stem cell collection	Azacitidine; hydroxyurea; “3 + 7 + midostaurin”	“3 + 7” FR; salvage HDAC + etoposide + idarubicin → CR	VCD + SCT; CyBorDex; KRd; RT; DRd
Immunotherapy/targeted	Nivolumab	No	Rituximab	No	No	Midostaurin	No	Daratumumab
Radiotherapy	SIRT	No	No	No	No	No	No	Yes
Experimental therapies	ICD, mEHT, NDV, WBH, IO-VAC	No	Hyperbaric	No	No	Topical amphotericin	Extensive surgery + local care	No
Other exposures	Dental procedures	—	Contact mucor patient	Multiple infections	RSV + prolonged hospitalization	—	Farm exposure	Travel
